# Prolactin rs1341239 T allele may have protective role against the brick tea type skeletal fluorosis

**DOI:** 10.1371/journal.pone.0171011

**Published:** 2017-02-02

**Authors:** Bing-Yun Li, Yan-Mei Yang, Yang Liu, Jing Sun, Yan Ye, Xiao-Na Liu, Hong-Xu Liu, Zhen-Qi Sun, Mang Li, Jing Cui, Dian-Jun Sun, Yan-Hui Gao

**Affiliations:** Center for Endemic Disease Control, Chinese Center for Disease Control and Prevention, Key Lab of Etiology and Epidemiology, Education Bureau of Heilongjiang Province & Ministry of Health (23618504), Harbin, Heilongjiang Province, China; Children's National Health System, UNITED STATES

## Abstract

**Objective:**

Prolactin (PRL) has been reported to be associated with increased bone turnover, and increased bone turnover is also a feature of skeletal fluorosis (SF). Autocrine/paracrine production of PRL is regulated by the extrapituitary promoter and a polymorphism in the extrapituitary PRL promoter at -1149 (rs1341239) is associated with disturbances of bone metabolism in other diseases. Here, we have investigated the possibility that the rs1341239 polymorphism is associated with SF, which results from the consumption of brick tea.

**Design:**

We conducted a cross-sectional study in Sinkiang, Qinghai, Inner Mongolia in China. Demography survey questionnaires were completed and physical examination and X-ray diagnoses were used to diagnose SF. Brick tea water fluoride intake (IF) and urinary fluoride (UF) were tested by an F-ion selective electrode method. A Sequenom MassARRAY system was used to determine PRL gene polymorphisms.

**Results:**

Subjects who were younger than 45 years of age and carried the T allele had a significantly decreased risk of SF [OR = 0.279 (95%CI, 0.094–0.824)] compared to those carrying the homozygous G allele. This phenomenon was only observed in Kazakh subjects [OR = 0.127 (95%CI, 0.025–0.646)]. Kazakh females who carried T alleles has a decreased risk of SF [OR = 0.410 (95%CI, 0.199–0.847)]. For Kazakh subjects which IF is less than 3.5 mg/d, a decreased risk of SF was observed among the participants who carried T alleles [OR = 0.118 (95%CI, 0.029–0.472)]. Overall, subjects with 1.6–3.2 mg/L UF and carried T alleles had a significantly decreased risk of SF [OR = 0.476 (95%CI, 0.237–0.955)] compared to homozygous G allele carriers. This phenomenon was only observed in Kazakh subjects [OR = 0.324 (95%CI, 0.114–0.923)].

**Conclusions:**

Our results suggested that the PRL rs1341239 T allele decreases the risk of brick tea SF.

## Introduction

The beneficial and detrimental effects of fluoride on human health are well established. A moderate amount of fluoride plays a critical role in preventing and controlling dental caries [1]. However, excessive exposure to fluoride can lead to skeletal fluorosis (SF) and dental fluorosis, which are caused by the disturbance of the bone metabolism and imbalances in enamel development, respectively. Fluorosis has been repeatedly documented in many field investigations and community surveys, for example, in Argentina, Brazil, Canada, China, Germany, India, South Africa, United Republic of Tanzania, and USA [2]. SF is more seriously than dental fluorosis as it causes pain and damage to bones and joints [3] and has the potential to cause permanent disability. The brick tea type fluorosis is prevalent in the northwestern area of China, including Tibet, Qinghai, Sinkiang, and Inner Mongolia, where members of minorities often drink brick tea (compressed black tea or green tea) with a high fluoride content, instead of drinking water [4–7]. It is important to note that 31 million people are affected [8]. Thus, it is crucial to explore new risk factors for fluorosis to improve prevention and treatment.

Fluoride toxicity depends on the following factors: (і) the total dose ingested, (ii) the duration of exposure, (iii) the nutritional status, and (iv) the body’s response. Among these factors, the amount of ingested fluoride is the major risk factor for fluorosis [9]. However, epidemiological and animal studies have shown individual differences in relation to the severity of dental fluorosis [10–14]. Single nucleotide polymorphisms (SNPs), which strongly correlate with disease susceptibility, have attracted researchers’ attention. Studies on children exposed to high concentrations of fluoride, report polymorphisms in the collagen type I alpha 2 or the estrogen receptor genes to be risk factors for dental fluorosis [12,15]. Our previous study showed that the G allele of the glutathione S-transferases pi class (GSTP1) gene rs1695 might be a protective factor against brick tea type SF [16]. SF is characterized by a high bone turnover, which may lead to bone sclerosis, bone softening, osteoporosis and heterotopic ossification. Therefore, genetic factors, especially SNPs, which may affect bone metabolism, may influence the pathogenesis of fluorosis.

Prolactin (PRL), produced by anterior pituitary gland [17] and various extrapituitary sites [18,19], is known to be associated with bone turnover. In addition to its classic function in mammary gland development and lactation, as PRL receptor is a member of cytokine receptor superfamily, PRL has been proposed to play multifunctional cytokine role [20]. Moreover, PRL is also synthesized and secreted by peripheral blood mononuclear cells (PBMCs) [21] and has been reported to promote lymphocytes proliferation, interacts with cytokines and functions as coactivator [22]. It is known that the expression of PRL is control by two different promoters: one controls the pituitary PRL production and the other controls extrapituitary prolactin production [23]. PRL -1149 G/T (rs1341239) polymorphism, which located in the extrapituitary promoter region, has been reported to regulate PRL mRNA expression in the T lymphocytes [24]. PRL rs1341239 alters protein-binding characteristics through binding to a GATA-related factor and PRL rs1341239 G allele generated higher levels of PRL in T lymphocytes [24]. It is worth noting that immune cells exert an indispensable role in regulating the activity of bone cells [25–28]. Moreover, multiple reports have shown PRL rs1341239 is related with rheumatoid arthritis (RA) or systemic lupus erythematosus (SLE), in which disturbance of bone metabolism is increased [19,22,24,29]. To sum up, the PRL rs1341239 may be associated with bone turnover.

Thus, we hypothesized that PRL rs1341239 polymorphic variations are potentially associated with brick tea SF. We conducted a cross-sectional study in three provinces (Sinkiang, Qinghai, Inner Mongolia) in China to investigate the relationship between PRL rs1341239 polymorphisms and SF.

## Materials and methods

### Subjects

We conducted a cross sectional investigation in sixteen villages from the Xinjiang, Qinghai, Inner Mongolia provinces in China in 2012. People above the age of 16 that had been living in these villages for more than 10 years were recruited. Demographic survey questionnaires were completed, and physical examination and X-rays were used to diagnose SF. The questionnaires included questions about name, age, sex, ethnicity, economic income, education, disease history, and brick tea drinking habits, including brick tea type, volumes consumed and frequency. A total of 859 participants were recruited and provided their water used to brew brick-tea, blood and urine for polymorphism analysis and fluoride determination.

The study was conducted under the approval of the ethical review board of Harbin Medical University (HMUIRB20120021). All subjects signed informed consent forms.

### Diagnosis of SF

SF was identified according to the Chinese Diagnostic Criteria of Endemic SF (WS192-2008, China) [30] using a diagnostic X-ray machine (Beijing Longsafe Imaging Technology Co., Beijing, China). Subjects without fluorosis were included in the controls group, and SF patients were allocated into the cases group. Three degrees of SF (mild, moderate and severe) were differentiated. Details on these categories can be found in our previous study [16].

### Assessment of water and urine samples

Urine samples and brick-tea water were stored at -20°C until detection. F-ion selective electrode (Yingke Crystal Materials Company, Hunan, China) was used by the Institute for Endemic Fluorosis Control at the Center for Endemic Disease Control (CDC) in China to analyze the fluoride concentration. All samples were measured in duplicate in two independent aliquots. The mean concentration between these two measurements represents the final fluoride concentration.

The analysis of the brick-tea water samples was performed according to a standardized method (GB19965-2005, China) [31]. The brick-tea water fluoride intake (IF) of all participants was classified into low exposure (less than 3.5 mg/d), intermediate exposure (3.5–7.0 mg/d) and high exposure (more than 7.0 mg/L). The urine samples were analyzed by a standardized method (WS/T89-2006, China) [32]. The urine fluoride (UF) concentration for all participants were categorized into the low load (less than 1.6 mg/L), intermediate-load (1.6–3.2 mg/L) and high-load (more than 3.2 mg/L).

### Identification of PRL polymorphisms and determination of genotypes

Genomic DNA was extracted using a whole blood DNA extraction kit (Axygen Biosciences, Union City, USA), and stored at -80°C for genotyping. DNA concentrations were measured by TU1901 spectrophotometry, which was purchased from Purkinje General Company (Beijing, China). Concentrations exceeded 20 μg/ml. Sequenom MassARRAY system purchased from Sequenom Inc. (San Diego, CA, USA) was used to determine the genetic sequence for genotyping. The sequence analysis was performed at the Shanghai Fenglin Clinical Laboratory [33]. The primer sequences for PRL rs1341239 were as follows: forward-5’-ACGTTGGATGCAGGTCAAGATAACCTGGAG-3’, reverse-5’-ACGTTGGATGATCACACTCAACCAGTTGGC-3’, extent-5’- AACCTGGAGAAAGGAGGAAAGA -3’.

Blinded blood duplicates were used for the sequencing quality control.

### Statistical analysis

The S1 Data was used as a database for this study. The statistical analysis was carried out using SPSS Statistics 19.0 (IBM). The significance level was set at 0.05. Differences between cases and controls were tested by Pearson’s chi-square test. Genotypes were tested for their Hardy-Weinberg equilibrium (HWE) among all controls or each ethnicity controls with a two-sided χ^2^ test. A binary logistic regression model was used to analyze the association of SNP variants and fluorosis condition. Fluorosis risk was calculated by computing odds ratios (ORs) and 95% confidence intervals (CI) with adjustment for age, sex, UF, IF or ethnicity.

## Results

### General characteristics

**Fig 1:**
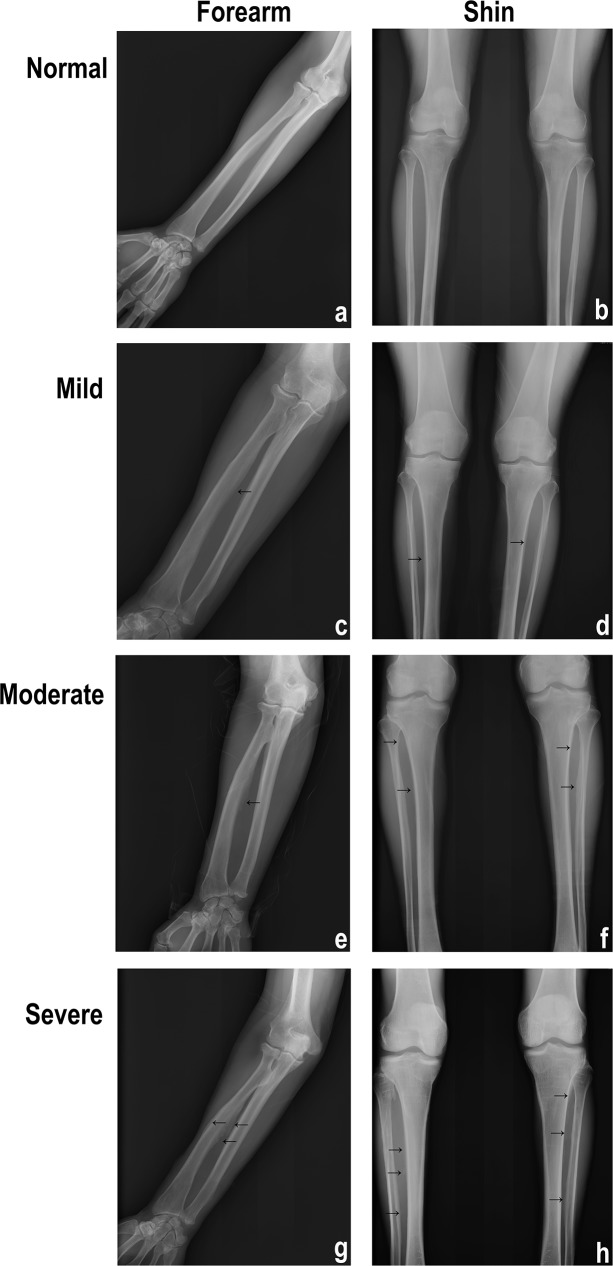
Representative images of brick-tea type skeletal fluorosis by X-ray examination. Normal (a) and (b): Normal forearm and shin. Mild (c) and (d): The interosseous membrane in the forearm or lower leg bone was mildly ossified with rough edges (arrows). Moderate (e) and (f): The radial crest and the border were enlarged; the interosseous membrane in the forearm or shin was ossified with serration or wave edge (arrows). Severe (g) and (h): The radial crest was enlarged and the border was obviously enlarged so that ulna and elbow joints presented severe degenerative changes; the interosseous membrane in the forearm or shin was obviously ossified with large shape (arrows).

X-ray diagnoses of cases showing different degrees of SF are presented in Fig 1. General characteristics of the 859 participants are presented in Table 1. Compared to the controls, more males were investigated in SF cases (p = 0.01), although we only observed this phenomenon in Tibetan subjects (p = 0.018). In Kazakh subjects more females were found in the SF cases, compared to the controls, but there is no significant difference (p = 0.066). Patients were significantly older than the controls (p<0.0001) and this phenomenon occurred in Tibetan subjects (p<0.0001), Kazakh subjects (p = 0.035) and Mongolian subjects (p = 0.017). SF patients ingested more fluoride (p = 0.007) and this phenomenon was also observed in Tibetan subjects (p = 0.011) and Mongolian subjects (p = 0.015). SF patients had a higher fluoride load (p = 0.001) as observed this phenomenon in Tibetan subjects (p = 0.013) and Mongolian subjects (p = 0.001). Moreover, we observed significant differences among three ethnical cases.

**Table 1:** General characteristics of study populations.

Characteristics	Cases				Controls				SF p*	Ethnicity p#
	Total n (%)	Tibetan n (%)	Kazakh n (%)	Mongolian n (%)	Total n (%)	Tibetan n (%)	Kazakh n (%)	Mongolian n (%)		
	279	123 (44.1)	98 (35.1)	58 (20.8)	580	185 (31.9)	192 (33.1)	203 (35.0)		
Sex									0.01	0.438
Male	130 (46.6)	64 (52.0)	44 (44.9)	22 (37.9)	217 (37.4)	71 (38.4)	65 (33.9)	81 (39.9)		
Female	149 (53.4)	59 (48.0)	54 (55.1)	36 (62.1)	363 (62.6)	114 (61.6)	127 (66.1)	122 (60.1)		
Age									<0.001	0.030
~45	54 (19.4)	20 (16.3)	20 (20.4)	14 (24.1)	235 (40.5)	78 (42.2)	66 (34.4)	91 (44.8)		
45~65	167 (59.9)	66 (53.7)	63 (64.3)	38 (65.5)	282 (48.6)	87 (47.0)	96 (50.0)	99 (48.8)		
65~	58 (20.8)	37 (30.1)	15 (15.3)	6 (10.3)	63 (10.9)	20 (10.8)	30 (15.6)	13 (6.4)		
IF									0.007	<0.001
~3.5 mg/d	78 (28.0)	27 (22.0)	23 (23.5)	28 (48.3)	168 (29.0)	41 (22.2)	45 (23.4)	82 (40.4)		
3.5~7.0 mg/d	88 (31.5)	33 (26.8)	37 (37.8)	18 (31.0)	236 (40.7)	78 (42.2)	57 (29.7)	101 (49.8)		
7.0 mg/d	113 (40.5)	63 (51.2)	38 (38.8)	12 (20.7)	176 (30.3)	66 (35.7)	90 (46.9)	20 (9.9)		
UF									<0.001	<0.001
~1.6 mg/L	64 (22.9)	21 (17.1)	18 (18.4)	25 (43.1)	206 (35.5)	59 (31.9)	24 (12.5)	123 (60.6)		
1.6~3.2 mg/L	109 (39.1)	59 (48.0)	32 (32.7)	18 (31.0)	204 (35.2)	69 (37.3)	72 (37.5)	63 (31.0)		
3.2 mg/L	106 (38.0)	43 (35.0)	48 (49.0)	15 (25.9)	170 (29.3)	57 (30.8)	96 (50.0)	17 (8.4)		
Grade of SF										<0.001
mild	197 (70.6)	68 (55.3)	82 (83.7)	47 (81.0)						
moderate	53 (19.0)	32 (26.0)	11 (11.2)	10 (17.2)						
severe	29 (10.4)	23 (18.7)	5 (5.10)	1 (1.7)						

p*, p value difference by fluorosis status; p#, p value difference by ethnicity (in case).

### Allele and genotype frequencies

All of the participants were found to be in HWE. The frequency of T allele homozygotes in rs1341239 was low and GT and TT were grouped together. According to the absence or presence of the T allele, GG or GT/TT groups were divided. Table 2 shows the occurrence of genotype and allele frequencies of the PRL rs1341239 polymorphism in three ethnical subjects. The genotype frequencies of PRL rs1341239 among three ethnical subjects showed significant differences (p<0.001).

**Table 2:** Occurrence of genotype and allele frequencies of the PRL rs1341239 polymorphism in three ethnical subjects.

Ethnicity	n	Genotype		HWE p	MAF %	p*
		GG	GT+TT			
Tibetan	308	280	28	0.403	4.55	0.001
Kazakh	290	185	105	0.942	20.17	
Mongolian	261	216	45	0.127	8.62	

N, number of subjects; HWE, Hardy-Weinberg equilibrium test; MAF, minor allele frequency; p*, significant difference by ethnicity.

### Association of PRL polymorphisms with skeletal fluorosis

The analysis of the potential association between genotypes and fluorosis is summarized in Table 3. Overall, we did not observe significant differences between cases with GG genotype and GT/TT genotype. After stratification by ethnicity, Kazakh subjects who carried the T allele had a tendency towards a decreased risk of SF (p = 0.051) in comparison to the GG genotype. Significant differences for the other two ethnic groups were not observed (Table 3).

**Table 3:** Associations between skeletal fluorosis and the PRL rs1341239 genotype (overall or stratified by ethnicity).

	Genotype	Case (n)	Control (n)	crude OR (95% CI)	adjusted OR (95% CI) ^a^
All subjects					
	GG	227	454	1 (ref)	1 (ref)
	GT+TT	52	126	1.212 (0.845–1.737)	1.167 (0.799–1.705)
Tibetan					
	GG	112	168	1 (ref)	1 (ref)
	GT+TT	11	17	0.971 (0.438–2.150)	1.198 (0.492–2.917)
Kazakh					
	GG	70	115	1 (ref)	1 (ref)
	GT+TT	28	77	0.597 (0.353–1.010)	**0.582 (0.337–1.006)** ^b^
Mongolian					
	GG	45	171	1 (ref)	1 (ref)
	GT+TT	13	32	1.544 (0.749–3.182)	1.506 (0.695–3.262)

a, Adjusted for age, gender, ethnicity, fluoride intake and urine fluoride.

b, p = 0.051.

We explored the potential for an interaction of rs1341239 and age (Table 4). For all of the subjects, a decreased risk of SF among carriers of the T allele was limited to ~45 group (p = 0.021). The phenomenon was only observed in Kazakh subjects (p = 0.013).

**Table 4:** Associations between skeletal fluorosis and the PRL rs1341239 genotype in different age groups (overall or stratified by ethnicity).

	Genotype	<45			45–60			>60		
		Case (n)	Control (n)	adjusted OR (95% CI)	Case (n)	Control (n)	adjusted OR (95% CI)	Case (n)	Control (n)	adjusted OR (95% CI)
All subjects										
	GG	50	187	1 (ref)	132	222	1 (ref)	45	45	1 (ref)
	GT+TT	4	48	**0.279 (0.094–0.824)** ^a^	35	60	1.180 (0.722–1.928)	13	18	0.974 (0.388–2.448)
Tibetan										
	GG	20	66		60	82	1 (ref)	32	20	
	GT+TT	0	12	——	6	5	1.87 (0.515–6.793)	5	0	——
Kazakh										
	GG	18	40	1 (ref)	43	63	1 (ref)	9	12	1 (ref)
	GT+TT	2	26	**0.127 (0.025–0.646)** ^b^	20	33	0.944 (0.473–1.885)	6	18	0.328 (0.065–1.646)
Mongolian										
	GG	12	81	1 (ref)	29	77	1 (ref)	4	13	1 (ref)
	GT+TT	2	10	1.054 (0.177–6.286)	9	22	1.185 (0.445–3.155)	2	0	1.147 (0.105–12.515)

Adjusted for gender, ethnicity, fluoride intake and urine fluoride.

a, p = 0.021

b, p = 0.013.

Moreover, we explored the associations between PRL rs1341239 genotype and gender (Table 5). No significant differences were detected for carrying the T allele. After stratified by ethnicity, a decreased risk of SF was observed among Kazakh female participants carrying T allele (p = 0.016).

**Table 5:** Associations between skeletal fluorosis and the PRL rs1341239 genotype in different gender groups (overall or stratified by ethnicity).

	Genotype	Females			Males		
		Case (n)	Control (n)	adjusted OR	Case (n)	Control (n)	adjusted OR
All subjects							
	GG	118	273	1(ref)	109	181	1(ref)
	GT+TT	31	90	0.770 (0.474–1250)	21	36	0.954 (0.511–1.780)
Tibetan							
	GG	54	104	1(ref)	58	64	1(ref)
	GT+TT	5	10	1.664 (0.467–5.931)	6	7	1.009 (0.284–3.593)
Kazakh							
	GG	39	69	1(ref)	31	46	1(ref)
	GT+TT	15	58	**0.410 (0.199–0.847)** ^**a**^	13	19	0.979 (0.4–2.396)
Mongolian							
	GG	25	100	1(ref)	20	71	1(ref)
	GT+TT	11	22	1.827 (0.710–4.703)	2	10	0.661 (0.128–3.425)

Adjusted for age, ethnicity, fluoride intake and urine fluoride.

a, p = 0.016.

Furthermore, we analyzed the interactions between PRL rs1341239 and fluoride exposure (Table 6). After stratification by IF, no significant differences were detected for carrying the T allele. Further stratification by ethnicity showed, a decreased risk of SF among Kazakh participants carrying T allele. This finding was limited to the low exposured group (p = 0.003).

**Table 6:** Associations between skeletal fluorosis and the PRL rs1341239 genotype in different IF groups (overall or stratified by ethnicity).

	Genotype	<3.5 mg/d			3.5–7.0 mg/d			>7 mg/d		
		Case (n)	Control (n)	adjusted OR (95% CI)	Case (n)	Control (n)	adjusted OR (95% CI)	Case (n)	Control (n)	adjusted OR (95% CI)
All subjects										
	GG	64	135	1 (ref)	70	181	1 (ref)	93	138	1 (ref)
	GT+TT	14	33	0.916 (0.504–1.662)	18	55	1.016 (0.624–1.655)	20	38	0.882 (0.475–1.636)
Tibetan										
	GG	22	41		31	68	1 (ref)	59	59	1 (ref)
	GT+TT	5	0	——	2	10	0.717 (0.129–3.998)	4	7	0.53 (0.127–2.218)
Kazakh										
	GG	20	21	1 (ref)	25	31	1 (ref)	25	63	1 (ref)
	GT+TT	3	24	**0.118 (0.029–0.472)** ^a^	12	26	0.459 (0.178–1.187)	13	27	1.686 (0.690–4.116)
Mongolian										
	GG	22	73	1 (ref)	14	82	1 (ref)	9	16	1 (ref)
	GT+TT	6	9	2.3 (0.717–7.381)	4	19	0.944 (0.255–3.495)	3	4	1.11 (0.107–11.565)

Adjusted for age, gender, ethnicity and urine fluoride.

a, p = 0.003.

The interactions between PRL rs1341239 and fluoride load were investigated (Table 7). For all of the subjects, a decreased risk of SF among carriers of the T allele was limited to the intermediate-load group (p = 0.035). This phenomenon was also observed in Kazakh subjects (p = 0.037).

**Table 7:** Associations between skeletal fluorosis and the PRL rs1341239 genotype in different UF groups (overall or stratified by ethnicity).

	Genotype	<1.6 mg/L			1.6–3.2 mg/L			>3.2 mg/L		
		Case (n)	Control (n)	adjusted OR	Case (n)	Control (n)	adjusted OR	Case (n)	Control (n)	adjusted OR
All subjects										
	GG	50	166	1 (ref)	96	158		81	130	
	GT+TT	14	40	1.154 (0.558–2.386)	13	46	**0.476 (0.237–0.955)** ^a^	25	40	1.107 (0.598–2.047)
Tibetan										
	GG	16	54	1 (ref)	57	61	1 (ref)	39	53	
	GT+TT	5	5	4.053 (0.893–18.398)	2	8	0.266 (0.047–1.525)	4	4	1.976 (0.360–10.848)
Kazakh										
	GG	13	12	1 (ref)	26	40	1 (ref)	31	63	
	GT+TT	5	12	0.299 (0.067–1.323)	6	32	**0.324 (0.114–0.923)** ^b^	17	33	1.100 (0.509–2.377)
Mongolian										
	GG	21	100	1 (ref)	13	57	1 (ref)	11	14	
	GT+TT	4	23	0.866 (0.255–2.942)	5	6	3.960 (0.961–16.315)	4	3	1.181 (0.170–8.194)

Adjusted for age, gender, ethnicity and fluoride intake.

a, p = 0.035

b, p = 0.037.

## Discussion

Brick-tea type fluorosis is a special type of fluorosis diffused in China, which is hard to prevent or eradicate, because it is almost impossible to change the habit of drinking brick tea water [34]. Excessive fluoride intake from brick tea water is the major risk factor for fluorosis. However, it could not explain the phenomenon that subjects showed different degrees of fluorosis when exposed to the same degree of fluoride [35,36]. Our previous study showed that the prevalence among five ethnical groups was different. The IF and UF were similar between Tibetan and Kazakh subjects, but Kazakh subjects had a lower prevalence rate [16]. These observations suggested that the individual genetic susceptibility to fluorosis would benefit from further research.

Because of the high bone turnover in fluorosis, we hypothesized that bone metabolism-related genes would be associated with the SF status. Several clinical studies had reported a possible role of PRL in bone development during pregnancy, lactation, or hyperprolactinemia [37–39]. Recently, human, mouse and rat studies have shown PRL receptor messenger RNA expression and intense of PRL receptor immunoreactivity appearance in various types of skeletal tissues during fetal development [40–45]. PRL receptors expressed in different tissues and osteoblasts suggested a possible direct action of PRL on bone [43,46]. Osteoblast-like cells, MG-63 and Saos-2, directly exposed to PRL exhibited lower expression of alkaline phosphatase and osteocalcin mRNA, and a decreased alkaline phosphatase activity [37,40]. Therefore, PRL could directly inhibit osteoblast function and bone formation. Otherwise, various lymphoid tissues and cells, including spleen, thymus, lymphocytes and macrophages, also express PRL and exert an antocrine action to promote lymphocytes proliferation. Interestingly, in normal immune state, the differentiation and activation of osteoclasts dependent on the nuclear factorκ B receptor activator ligand (RANKL) secreted by osteoblasts binding to nuclear factorκ B receptor activator (RANK) [47]. Nevertheless, in enabled immune system, activated T lymphocytes could express RANKL to affect osteoclasts via the same pathway as previous discription [48,49]. In summary, PRL may influence on bone metabolism.

The PRL rs1341239 SNP is located within 2 kb upstream of PRL, the extrapituitary promoter region, and might have an influence on PRL mRNA expression [22]. This SNP has been found to be correlated to RA and SLE. One study, which included 173 rheumatoid arthritis patients and 123 healthy controls, found evidence that the heterozygous genotype of PRL rs1341239 was associated with increased rheumatoid arthritis risk [19]. Another large study assembled a cohort of 3,405 cases and 4,111 controls, and suggested an association between the PRL rs1341239 T allele and decreased rheumatoid arthritis risk [24]. In the present study, we observed that the genotype frequencies of PRL rs1341239 were different among different ethnical subjects as follows: The T allele (minor) was more common among Kazakh subjects. It is mentioned in our previous study that the prevalence rate of SF in Kazakh subjects is less than that in Tibetan subjects [16]. In the current study, Kazakh participants who carried the T allele had a slightly decreased risk of SF compared to GG genotype, but this tendency was not observed in other ethnic participants with the same allele. In addition, the PRL rs1341239 SNP has been linked to RA and SLE, which are known to be female predominant. So, we explored the associations between PRL rs1341239 genotype and gender. The protective effect of the T allele in PRL rs1341239 against brick-tea type SF was only observed among Kazakh female participants. The genotype frequencies of PRL rs1341239 between female and male Kazakh subjects showed significant differences (p = 0.039): the T allele (minor) was more common among female Kazakh subjects. Moreover, in our study less females Kazakh subjects were found in the SF cases, compared to the controls, although there is no significant difference (p = 0.066). These results suggest that the protective of the T allele in PRL rs1341239 against brick-tea type SF might be present among female Kazakh subjects. However, the female patient from other origins carried the same allele did not present resistance by the statistical analysis. These results suggest that the protective role of the T allele against SF might interfere with fluoride exposure and sample size.

Dose and duration of fluoride exposure are the main risk factors related to variations in the clinical presentation [9]. Thus, we further investigated the potential interaction between the PRL rs1341239 SNP and known risk factors or ethnic. Stratification by age or by fluoride load showed that the protective role of the T allele in PRL rs1341239 against SF appeared to be more prominent for subjects aged less than 45 years old or in the intermediate-load group. This was attributed to the reduced risk in Kazakh subjects. Furthermore, the protective effect of the T allele in PRL rs1341239 against brick-tea type SF was detected in Kazakh subjects with less than 3.5 mg/d IF. In conclusion, the protective role of the T allele in PRL rs1341239 against brick-tea type SF appeared to be prominent in the subjects with low fluoride exposure and short exposure duration, especially for Kazakh subjects. However, when these research factors were analyzed simultaneously in other two ethnics, the data became too sparse. Therefore, these findings on the PRL rs1341239 interaction with the risk factors need to be interpreted with deliberation.

In summary, our results suggest that the T allele in PRL rs1341239 decreased the risk of brick tea type SF, especially in female Kazakh subjects. In consideration of the sample size after stratification, a larger investigation should be conducted to obtain more information. In addition, future studies are necessary to clarify the functional role and mechanism of the T allele in PRL rs1341239 on brick tea SF.

## Supporting information

S1 Data Data of the study (PDF). (PDF)
