# Supplementary material for: Prolactin rs1341239 T allele may have protective role against the brick tea type skeletal fluorosis
Source: PLoS One. 2017 Feb 2;12(2):e0171011. doi: 10.1371/journal.pone.0171011 (PMC5289533; doi:10.1371/journal.pone.0171011)
Supplement: S1 Data — (PDF) [file pone.0171011.s001.pdf]

area	number	sex	age	race	urine fluoride (mg/L)	intake of fluoride per day (mg/d)	skeletal fluorosis	skeletal fluorosis grade	PRL rs1341239
Inner Mongolia	1	Male	23	Mongolian	1.228	4.402	No	No	GG
Inner Mongolia	2	Male	25	Mongolian	1.636	4.957	No	No	GG
Inner Mongolia	3	Male	26	Mongolian	1.220	3.320	No	No	GG
Inner Mongolia	4	Male	26	Mongolian	1.276	4.468	No	No	GG
Inner Mongolia	5	Male	26	Mongolian	1.181	4.339	No	No	GG
Inner Mongolia	6	Male	26	Mongolian	2.523	6.160	No	No	GG
Inner Mongolia	7	Female	27	Mongolian	1.017	4.116	No	No	GT
Inner Mongolia	8	Male	27	Mongolian	0.779	3.794	No	No	GG
Inner Mongolia	9	Male	27	Mongolian	3.200	7.078	No	No	GG
Inner Mongolia	10	Female	28	Mongolian	0.423	3.310	No	No	GG
Inner Mongolia	11	Female	28	Mongolian	1.101	4.231	No	No	GG
Inner Mongolia	12	Male	28	Mongolian	7.394	12.769	No	No	GG
Inner Mongolia	13	Male	29	Mongolian	4.883	9.362	Yes	Mild	GG
Inner Mongolia	14	Male	30	Mongolian	1.170	4.325	No	No	GG
Inner Mongolia	15	Female	30	Mongolian	2.606	2.674	No	No	GG
Inner Mongolia	16	Female	30	Mongolian	0.756	3.763	No	No	GG
Inner Mongolia	17	Male	30	Mongolian	1.182	4.341	No	No	GG
Inner Mongolia	18	Female	31	Mongolian	0.698	3.684	No	No	GG
Inner Mongolia	19	Female	31	Mongolian	0.867	2.344	No	No	GT
Inner Mongolia	20	Female	32	Mongolian	1.341	4.557	No	No	GG
Inner Mongolia	21	Male	32	Mongolian	0.831	13.071	No	No	GT
Inner Mongolia	22	Female	32	Mongolian	1.136	4.024	No	No	GG
Inner Mongolia	23	Female	32	Mongolian	0.850	2.592	Yes	Mild	GG
Inner Mongolia	24	Male	32	Mongolian	4.694	9.106	No	No	GG

Inner Mongolia	25	Female	33	Mongolian	1.164	4.316	No	No	GG
Inner Mongolia	26	Female	33	Mongolian	0.463	3.364	No	No	GG
Inner Mongolia	27	Male	33	Mongolian	3.818	7.917	No	No	GG
Inner Mongolia	28	Female	34	Mongolian	0.636	0.400	No	No	GG
Inner Mongolia	29	Male	34	Mongolian	1.779	2.592	Yes	Mild	GG
Inner Mongolia	30	Female	34	Mongolian	2.389	0.957	No	No	GG
Inner Mongolia	31	Female	34	Mongolian	1.965	5.402	No	No	GG
Inner Mongolia	32	Female	35	Mongolian	2.389	5.978	Yes	Mild	GG
Inner Mongolia	33	Male	36	Mongolian	0.758	3.765	No	No	GT
Inner Mongolia	34	Female	36	Mongolian	0.636	1.163	No	No	GG
Inner Mongolia	35	Female	37	Mongolian	0.555	3.489	No	No	GG
Inner Mongolia	36	Female	37	Mongolian	5.085	9.636	No	No	GG
Inner Mongolia	37	Female	37	Mongolian	1.326	4.536	No	No	GG
Inner Mongolia	38	Male	37	Mongolian	0.592	3.541	No	No	GG
Inner Mongolia	39	Male	37	Mongolian	1.406	0.851	Yes	Mild	GG
Inner Mongolia	40	Female	38	Mongolian	2.737	5.336	Yes	Mild	GG
Inner Mongolia	41	Female	39	Mongolian	1.307	4.510	No	No	GG
Inner Mongolia	42	Female	39	Mongolian	0.909	3.970	No	No	GG
Inner Mongolia	43	Female	39	Mongolian	0.493	3.507	No	No	GG
Inner Mongolia	44	Male	39	Mongolian	3.265	7.167	No	No	GG
Inner Mongolia	45	Female	40	Mongolian	1.212	1.753	No	No	GG
Inner Mongolia	46	Male	40	Mongolian	0.732	3.730	No	No	GG
Inner Mongolia	47	Female	40	Mongolian	1.231	7.806	No	No	GT
Inner Mongolia	48	Male	40	Mongolian	2.641	12.331	No	No	GG
Inner Mongolia	49	Female	40	Mongolian	0.634	1.158	No	No	GG
Inner Mongolia	50	Female	40	Mongolian	0.485	0.355	Yes	Mild	GG

Inner Mongolia	51	Female	40	Mongolian	1.276	4.024	Yes	Mild	GG
Inner Mongolia	52	Female	40	Mongolian	0.820	3.850	No	No	GG
Inner Mongolia	53	Female	40	Mongolian	1.617	4.930	No	No	GG
Inner Mongolia	54	Female	40	Mongolian	0.965	4.046	No	No	GG
Inner Mongolia	55	Female	40	Mongolian	0.518	3.440	No	No	GG
Inner Mongolia	56	Male	40	Mongolian	6.598	11.689	No	No	GT
Inner Mongolia	57	Female	40	Mongolian	1.329	4.483	No	No	GG
Inner Mongolia	58	Male	40	Mongolian	1.783	2.989	No	No	GG
Inner Mongolia	59	Female	41	Mongolian	1.457	0.212	No	No	GG
Inner Mongolia	60	Male	41	Mongolian	2.715	5.204	No	No	GG
Inner Mongolia	61	Female	41	Mongolian	2.300	4.099	No	No	GG
Inner Mongolia	62	Female	41	Mongolian	1.017	4.116	No	No	GG
Inner Mongolia	63	Male	41	Mongolian	1.429	4.676	No	No	GG
Inner Mongolia	64	Female	41	Mongolian	0.658	3.629	No	No	GG
Inner Mongolia	65	Female	42	Mongolian	2.954	6.745	No	No	GG
Inner Mongolia	66	Male	42	Mongolian	2.100	0.877	No	No	GG
Inner Mongolia	67	Male	42	Mongolian	1.616	4.929	No	No	GG
Inner Mongolia	68	Female	42	Mongolian	1.350	1.753	No	No	GG
Inner Mongolia	69	Female	42	Mongolian	0.694	0.877	No	No	GG
Inner Mongolia	70	Female	42	Mongolian	1.466	4.725	No	No	GG
Inner Mongolia	71	Male	42	Mongolian	0.540	3.470	No	No	GG
Inner Mongolia	72	Male	42	Mongolian	1.360	4.582	No	No	GG
Inner Mongolia	73	Female	42	Mongolian	1.406	0.544	No	No	GG
Inner Mongolia	74	Male	42	Mongolian	0.378	3.250	No	No	GG
Inner Mongolia	75	Female	42	Mongolian	0.402	3.282	Yes	Mild	GG
Inner Mongolia	76	Male	42	Mongolian	1.495	4.766	No	No	GG

Inner Mongolia	77	Female	42	Mongolian	2.124	5.619	No	No	GG
Inner Mongolia	78	Female	43	Mongolian	1.044	4.153	No	No	GG
Inner Mongolia	79	Female	43	Mongolian	1.399	3.507	No	No	GG
Inner Mongolia	80	Female	43	Mongolian	2.756	21.969	No	No	GG
Inner Mongolia	81	Female	43	Mongolian	2.119	5.612	No	No	GG
Inner Mongolia	82	Male	43	Mongolian	0.435	3.327	No	No	GG
Inner Mongolia	83	Female	43	Mongolian	0.500	3.415	No	No	GT
Inner Mongolia	84	Male	43	Mongolian	2.043	5.509	No	No	GG
Inner Mongolia	85	Male	43	Mongolian	3.818	7.917	No	No	GG
Inner Mongolia	86	Female	44	Mongolian	1.349	4.567	No	No	GT
Inner Mongolia	87	Female	44	Mongolian	3.923	0.354	Yes	Mild	GT
Inner Mongolia	88	Female	44	Mongolian	0.868	0.091	Yes	Mild	GG
Inner Mongolia	89	Female	44	Mongolian	0.447	0.122	No	No	GG
Inner Mongolia	90	Female	44	Mongolian	1.128	0.877	No	No	GT
Inner Mongolia	91	Female	44	Mongolian	4.410	15.854	No	No	GG
Inner Mongolia	92	Female	44	Mongolian	1.655	1.921	No	No	GG
Inner Mongolia	93	Female	44	Mongolian	0.524	1.554	No	No	GG
Inner Mongolia	94	Female	44	Mongolian	0.515	1.127	Yes	Mild	GG
Inner Mongolia	95	Female	44	Mongolian	1.406	0.298	No	No	GT
Inner Mongolia	96	Male	44	Mongolian	1.087	4.211	No	No	GG
Inner Mongolia	97	Female	44	Mongolian	0.729	2.110	No	No	GG
Inner Mongolia	98	Male	45	Mongolian	1.406	0.236	No	No	GG
Inner Mongolia	99	Female	45	Mongolian	2.179	2.965	Yes	Mild	GT
Inner Mongolia	100	Male	45	Mongolian	0.968	4.051	No	No	GG
Inner Mongolia	101	Female	45	Mongolian	1.200	4.365	No	No	GG
Inner Mongolia	102	Female	45	Mongolian	0.440	0.947	No	No	GG

Inner Mongolia	103	Female	45	Mongolian	1.965	0.928	No	No	GG
Inner Mongolia	104	Male	45	Mongolian	5.607	10.345	Yes	Mild	GG
Inner Mongolia	105	Female	45	Mongolian	5.868	10.699	No	No	GG
Inner Mongolia	106	Female	46	Mongolian	1.146	4.292	No	No	GT
Inner Mongolia	107	Female	46	Mongolian	1.162	4.313	No	No	GT
Inner Mongolia	108	Male	46	Mongolian	2.400	5.993	No	No	GG
Inner Mongolia	109	Female	46	Mongolian	3.761	7.839	No	No	GG
Inner Mongolia	110	Female	46	Mongolian	1.716	4.431	No	No	GT
Inner Mongolia	111	Male	46	Mongolian	3.570	2.965	No	No	GG
Inner Mongolia	112	Female	46	Mongolian	0.738	3.738	No	No	GT
Inner Mongolia	113	Female	46	Mongolian	3.671	7.718	No	No	GT
Inner Mongolia	114	Female	46	Mongolian	1.437	4.687	No	No	GG
Inner Mongolia	115	Female	46	Mongolian	5.412	10.079	Yes	Moderate	GG
Inner Mongolia	116	Female	47	Mongolian	1.350	1.534	Yes	Mild	GG
Inner Mongolia	117	Female	47	Mongolian	0.733	1.465	No	No	GG
Inner Mongolia	118	Male	47	Mongolian	1.529	6.038	No	No	GG
Inner Mongolia	119	Female	47	Mongolian	1.171	0.700	No	No	GG
Inner Mongolia	120	Female	47	Mongolian	1.240	0.463	No	No	GG
Inner Mongolia	121	Female	47	Mongolian	0.415	3.300	No	No	GG
Inner Mongolia	122	Female	47	Mongolian	0.767	3.777	No	No	GG
Inner Mongolia	123	Female	47	Mongolian	2.418	6.017	No	No	GG
Inner Mongolia	124	Male	47	Mongolian	1.395	4.630	No	No	GG
Inner Mongolia	125	Male	47	Mongolian	1.229	4.404	No	No	GT
Inner Mongolia	126	Male	48	Mongolian	1.616	1.753	No	No	GG
Inner Mongolia	127	Male	48	Mongolian	1.044	5.145	No	No	GT
Inner Mongolia	128	Female	48	Mongolian	0.544	3.475	Yes	Mild	GT

Inner Mongolia	129	Female	48	Mongolian	1.509	1.689	Yes	Mild	GT
Inner Mongolia	130	Female	48	Mongolian	0.661	1.569	No	No	GG
Inner Mongolia	131	Male	48	Mongolian	0.619	1.277	Yes	Mild	GG
Inner Mongolia	132	Female	48	Mongolian	2.485	6.108	No	No	GG
Inner Mongolia	133	Female	48	Mongolian	2.361	5.939	No	No	GG
Inner Mongolia	134	Female	48	Mongolian	6.345	11.346	No	No	GG
Inner Mongolia	135	Male	49	Mongolian	14.619	1.752	No	No	GG
Inner Mongolia	136	Female	49	Mongolian	1.620	4.935	Yes	Mild	GG
Inner Mongolia	137	Male	49	Mongolian	1.406	0.589	No	No	GT
Inner Mongolia	138	Female	49	Mongolian	4.011	15.854	Yes	Moderate	GT
Inner Mongolia	139	Female	49	Mongolian	2.179	5.693	No	No	GG
Inner Mongolia	140	Male	49	Mongolian	0.834	1.569	Yes	Moderate	GG
Inner Mongolia	141	Male	49	Mongolian	0.834	3.868	No	No	GG
Inner Mongolia	142	Female	49	Mongolian	2.370	5.952	No	No	GT
Inner Mongolia	143	Female	49	Mongolian	1.181	0.269	No	No	GG
Inner Mongolia	144	Male	49	Mongolian	1.412	4.652	No	No	GG
Inner Mongolia	145	Female	49	Mongolian	2.732	6.443	No	No	GT
Inner Mongolia	146	Male	49	Mongolian	1.437	4.687	Yes	Severe	GG
Inner Mongolia	147	Female	49	Mongolian	5.643	10.394	Yes	Mild	GG
Inner Mongolia	148	Male	50	Mongolian	1.951	1.753	Yes	Mild	GG
Inner Mongolia	149	Female	50	Mongolian	1.265	4.452	No	No	GG
Inner Mongolia	150	Male	50	Mongolian	1.951	5.384	No	No	GG
Inner Mongolia	151	Male	50	Mongolian	0.705	0.438	No	No	GG
Inner Mongolia	152	Male	50	Mongolian	0.744	1.315	No	No	GG
Inner Mongolia	153	Male	50	Mongolian	1.562	0.351	No	No	GT
Inner Mongolia	154	Female	50	Mongolian	1.132	4.272	Yes	Mild	GG

Inner Mongolia	155	Female	50	Mongolian	2.179	1.349	No	No	GG
Inner Mongolia	156	Female	50	Mongolian	0.936	4.007	Yes	Mild	GT
Inner Mongolia	157	Female	50	Mongolian	1.406	0.050	No	No	GG
Inner Mongolia	158	Female	50	Mongolian	1.624	4.759	No	No	GT
Inner Mongolia	159	Male	50	Mongolian	1.888	5.299	No	No	GT
Inner Mongolia	160	Female	50	Mongolian	1.908	5.325	Yes	Mild	GG
Inner Mongolia	161	Female	50	Mongolian	2.056	0.672	No	No	GG
Inner Mongolia	162	Female	50	Mongolian	2.269	5.815	No	No	GG
Inner Mongolia	163	Female	51	Mongolian	1.305	4.507	No	No	GG
Inner Mongolia	164	Female	51	Mongolian	0.749	1.753	No	No	GG
Inner Mongolia	165	Female	51	Mongolian	1.141	4.285	No	No	GT
Inner Mongolia	166	Female	51	Mongolian	3.440	2.708	Yes	Mild	GG
Inner Mongolia	167	Female	51	Mongolian	1.996	5.445	No	No	GG
Inner Mongolia	168	Male	51	Mongolian	1.465	4.715	Yes	Mild	GG
Inner Mongolia	169	Male	51	Mongolian	2.445	5.081	No	No	GT
Inner Mongolia	170	Female	51	Mongolian	1.270	1.055	Yes	Mild	GG
Inner Mongolia	171	Male	51	Mongolian	1.772	5.141	Yes	Mild	GG
Inner Mongolia	172	Male	51	Mongolian	1.030	4.134	No	No	GG
Inner Mongolia	173	Male	51	Mongolian	2.647	6.328	No	No	GG
Inner Mongolia	174	Female	51	Mongolian	3.355	7.289	Yes	Mild	GG
Inner Mongolia	175	Female	52	Mongolian	2.258	0.174	No	No	GG
Inner Mongolia	176	Female	52	Mongolian	4.795	2.484	No	No	GT
Inner Mongolia	177	Female	52	Mongolian	1.316	4.523	No	No	GG
Inner Mongolia	178	Female	52	Mongolian	1.911	2.420	Yes	Mild	GG
Inner Mongolia	179	Male	52	Mongolian	1.911	5.330	No	No	GG
Inner Mongolia	180	Female	52	Mongolian	1.602	2.505	No	No	GG

Inner Mongolia	181	Male	52	Mongolian	2.085	1.487	No	No	GG
Inner Mongolia	182	Female	52	Mongolian	4.827	9.286	Yes	Moderate	GG
Inner Mongolia	183	Female	53	Mongolian	0.925	3.991	No	No	GT
Inner Mongolia	184	Female	53	Mongolian	1.472	1.320	No	No	GG
Inner Mongolia	185	Male	53	Mongolian	2.509	2.062	No	No	GG
Inner Mongolia	186	Male	53	Mongolian	1.839	5.232	No	No	GG
Inner Mongolia	187	Male	53	Mongolian	2.223	5.752	No	No	GG
Inner Mongolia	188	Male	53	Mongolian	0.860	3.904	No	No	GG
Inner Mongolia	189	Male	53	Mongolian	3.762	0.672	Yes	Mild	GG
Inner Mongolia	190	Male	53	Mongolian	3.463	1.278	No	No	GG
Inner Mongolia	191	Female	53	Mongolian	1.382	1.278	No	No	GG
Inner Mongolia	192	Male	53	Mongolian	3.175	3.757	Yes	Mild	GT
Inner Mongolia	193	Male	54	Mongolian	1.256	4.441	No	No	GT
Inner Mongolia	194	Male	54	Mongolian	2.620	1.753	No	No	GG
Inner Mongolia	195	Male	54	Mongolian	1.752	3.280	Yes	Mild	GG
Inner Mongolia	196	Male	54	Mongolian	1.127	4.266	Yes	Mild	GG
Inner Mongolia	197	Female	54	Mongolian	1.650	0.619	Yes	Mild	GT
Inner Mongolia	198	Female	54	Mongolian	0.619	1.114	Yes	Mild	GG
Inner Mongolia	199	Male	54	Mongolian	1.296	0.167	Yes	Mild	GG
Inner Mongolia	200	Male	54	Mongolian	9.521	10.850	No	No	GG
Inner Mongolia	201	Female	55	Mongolian	0.819	0.136	No	No	GG
Inner Mongolia	202	Male	55	Mongolian	1.666	4.996	No	No	GG
Inner Mongolia	203	Female	55	Mongolian	1.866	0.614	No	No	GG
Inner Mongolia	204	Female	55	Mongolian	0.932	1.033	No	No	GT
Inner Mongolia	205	Male	55	Mongolian	2.365	5.945	No	No	GG
Inner Mongolia	206	Female	55	Mongolian	3.661	7.704	Yes	Mild	GT

Inner Mongolia	207	Male	55	Mongolian	1.317	4.524	No	No	GG
Inner Mongolia	208	Female	55	Mongolian	0.383	3.256	No	No	GT
Inner Mongolia	209	Female	55	Mongolian	0.557	1.699	Yes	Mild	GG
Inner Mongolia	210	Female	55	Mongolian	2.992	6.796	Yes	Mild	GT
Inner Mongolia	211	Female	56	Mongolian	1.550	3.037	No	No	GG
Inner Mongolia	212	Female	56	Mongolian	1.093	4.220	No	No	GG
Inner Mongolia	213	Female	56	Mongolian	2.767	6.491	No	No	GG
Inner Mongolia	214	Female	56	Mongolian	1.736	5.092	No	No	GG
Inner Mongolia	215	Female	56	Mongolian	0.978	4.064	No	No	GG
Inner Mongolia	216	Male	56	Mongolian	1.365	9.518	No	No	GG
Inner Mongolia	217	Female	57	Mongolian	0.916	3.980	No	No	GT
Inner Mongolia	218	Female	57	Mongolian	1.216	4.839	No	No	GT
Inner Mongolia	219	Male	57	Mongolian	1.406	0.589	No	No	GG
Inner Mongolia	220	Male	57	Mongolian	2.112	5.602	No	No	GG
Inner Mongolia	221	Female	57	Mongolian	1.920	0.956	No	No	GG
Inner Mongolia	222	Male	57	Mongolian	4.642	8.526	Yes	Moderate	GG
Inner Mongolia	223	Female	57	Mongolian	2.505	8.526	Yes	Moderate	GG
Inner Mongolia	224	Male	58	Mongolian	2.445	2.357	No	No	GG
Inner Mongolia	225	Male	58	Mongolian	2.965	15.854	Yes	Mild	GG
Inner Mongolia	226	Female	58	Mongolian	1.808	5.189	Yes	Mild	GG
Inner Mongolia	227	Male	59	Mongolian	0.320	3.171	No	No	GG
Inner Mongolia	228	Female	59	Mongolian	1.762	5.127	No	No	GG
Inner Mongolia	229	Male	59	Mongolian	0.688	3.670	No	No	GG
Inner Mongolia	230	Female	59	Mongolian	1.839	2.945	No	No	GG
Inner Mongolia	231	Female	59	Mongolian	3.715	1.212	Yes	Mild	GG
Inner Mongolia	232	Female	60	Mongolian	1.588	0.877	No	No	GG

Inner Mongolia	233	Female	60	Mongolian	1.045	1.212	No	No	GG
Inner Mongolia	234	Male	60	Mongolian	1.250	4.433	Yes	Mild	GG
Inner Mongolia	235	Female	61	Mongolian	0.506	1.258	No	No	GG
Inner Mongolia	236	Male	61	Mongolian	0.713	3.704	No	No	GG
Inner Mongolia	237	Female	61	Mongolian	3.641	7.677	Yes	Moderate	GT
Inner Mongolia	238	Male	62	Mongolian	2.030	5.491	No	No	GG
Inner Mongolia	239	Female	62	Mongolian	0.845	0.252	No	No	GG
Inner Mongolia	240	Female	63	Mongolian	0.643	2.630	No	No	GG
Inner Mongolia	241	Female	64	Mongolian	2.085	1.179	No	No	GG
Inner Mongolia	242	Female	65	Mongolian	0.731	3.728	No	No	GG
Inner Mongolia	243	Male	66	Mongolian	0.447	2.192	No	No	GG
Inner Mongolia	244	Male	66	Mongolian	1.260	0.764	Yes	Moderate	GG
Inner Mongolia	245	Female	68	Mongolian	1.529	0.764	Yes	Moderate	GT
Inner Mongolia	246	Male	68	Mongolian	1.172	4.327	Yes	Mild	GG
Inner Mongolia	247	Female	68	Mongolian	2.941	6.727	No	No	GG
Inner Mongolia	248	Male	69	Mongolian	2.521	0.531	No	No	GG
Inner Mongolia	249	Female	72	Mongolian	1.049	0.614	No	No	GG
Inner Mongolia	250	Female	72	Mongolian	0.939	4.010	No	No	GG
Inner Mongolia	251	Female	74	Mongolian	1.589	3.459	No	No	GG
Inner Mongolia	252	Male	74	Mongolian	1.624	0.485	No	No	GG
Inner Mongolia	253	Female	75	Mongolian	6.299	2.325	Yes	Mild	GG
Inner Mongolia	254	Female	75	Mongolian	1.406	0.648	No	No	GG
Inner Mongolia	255	Male	75	Mongolian	0.449	0.648	No	No	GG
Inner Mongolia	256	Male	75	Mongolian	1.650	4.975	Yes	Mild	GG
Inner Mongolia	257	Female	76	Mongolian	1.428	4.674	No	No	GG
Inner Mongolia	258	Male	76	Mongolian	1.714	4.586	Yes	Moderate	GT

Inner Mongolia	259	Male	78	Mongolian	0.485	3.395	No	No	GG
Inner Mongolia	260	Female	78	Mongolian	1.889	8.526	No	No	GG
Inner Mongolia	261	Male	80	Mongolian	2.542	1.627	No	No	GG
Qinghai	262	Male	24	Tibetan	1.200	4.364	No	No	GT
Qinghai	263	Female	26	Tibetan	0.944	2.051	No	No	GG
Qinghai	264	Female	28	Tibetan	2.085	1.127	No	No	GG
Qinghai	265	Female	29	Tibetan	1.769	5.136	No	No	GG
Qinghai	266	Male	30	Tibetan	0.819	3.848	No	No	GG
Qinghai	267	Male	30	Tibetan	7.888	13.440	No	No	GG
Qinghai	268	Male	30	Tibetan	3.200	6.000	No	No	GG
Qinghai	269	Female	30	Tibetan	0.808	8.465	No	No	GG
Qinghai	270	Female	31	Tibetan	3.591	3.075	No	No	GG
Qinghai	271	Female	32	Tibetan	1.079	4.200	No	No	GG
Qinghai	272	Female	32	Tibetan	2.088	6.410	No	No	GG
Qinghai	273	Female	32	Tibetan	0.502	2.410	No	No	GG
Qinghai	274	Female	33	Tibetan	0.470	3.375	No	No	GG
Qinghai	275	Female	33	Tibetan	0.727	3.723	No	No	GG
Qinghai	276	Female	33	Tibetan	1.406	0.732	No	No	GG
Qinghai	277	Female	33	Tibetan	2.571	5.136	No	No	GT
Qinghai	278	Female	34	Tibetan	0.940	3.532	No	No	GG
Qinghai	279	Female	34	Tibetan	2.130	3.465	No	No	GG
Qinghai	280	Female	35	Tibetan	7.345	12.703	Yes	Mild	GG
Qinghai	281	Female	35	Tibetan	1.918	5.338	Yes	Moderate	GG
Qinghai	282	Female	35	Tibetan	0.481	3.261	No	No	GG
Qinghai	283	Female	36	Tibetan	1.801	1.664	No	No	GG
Qinghai	284	Female	36	Tibetan	2.574	5.838	No	No	GG

Qinghai	285	Female	36	Tibetan	0.620	3.578	No	No	GG
Qinghai	286	Male	36	Tibetan	1.768	2.173	No	No	GG
Qinghai	287	Female	37	Tibetan	0.751	3.756	No	No	GG
Qinghai	288	Male	37	Tibetan	1.567	4.863	No	No	GG
Qinghai	289	Female	37	Tibetan	3.531	7.528	No	No	GT
Qinghai	290	Male	37	Tibetan	2.582	6.240	No	No	GG
Qinghai	291	Female	37	Tibetan	1.361	13.303	No	No	GG
Qinghai	292	Female	37	Tibetan	1.219	26.333	No	No	GG
Qinghai	293	Male	37	Tibetan	3.422	7.379	No	No	GT
Qinghai	294	Female	37	Tibetan	1.795	12.714	No	No	GG
Qinghai	295	Female	37	Tibetan	1.771	23.721	No	No	GG
Qinghai	296	Female	38	Tibetan	2.997	1.266	No	No	GG
Qinghai	297	Male	38	Tibetan	1.813	5.196	No	No	GG
Qinghai	298	Female	38	Tibetan	1.135	4.276	No	No	GG
Qinghai	299	Female	38	Tibetan	2.378	1.029	No	No	GG
Qinghai	300	Male	38	Tibetan	0.921	3.986	No	No	GG
Qinghai	301	Female	38	Tibetan	1.767	1.809	Yes	Mild	GG
Qinghai	302	Female	38	Tibetan	3.664	7.708	No	No	GG
Qinghai	303	Female	38	Tibetan	0.963	11.217	No	No	GT
Qinghai	304	Female	38	Tibetan	1.184	8.617	No	No	GG
Qinghai	305	Female	38	Tibetan	0.746	2.632	No	No	GG
Qinghai	306	Female	39	Tibetan	16.608	25.270	No	No	GG
Qinghai	307	Female	39	Tibetan	0.856	0.541	No	No	GG
Qinghai	308	Female	39	Tibetan	0.996	4.088	No	No	GG
Qinghai	309	Male	39	Tibetan	1.854	5.253	No	No	GG
Qinghai	310	Male	39	Tibetan	2.998	6.805	No	No	GG

Qinghai	311	Female	39	Tibetan	0.710	3.701	No	No	GT
Qinghai	312	Female	39	Tibetan	2.916	8.050	Yes	Mild	GG
Qinghai	313	Male	39	Tibetan	1.868	0.564	Yes	Moderate	GG
Qinghai	314	Female	39	Tibetan	2.478	6.099	No	No	GG
Qinghai	315	Female	40	Tibetan	5.855	10.681	No	No	GG
Qinghai	316	Male	40	Tibetan	3.505	8.787	No	No	GG
Qinghai	317	Female	40	Tibetan	1.495	9.045	Yes	Mild	GG
Qinghai	318	Female	40	Tibetan	6.214	2.851	No	No	GG
Qinghai	319	Male	40	Tibetan	3.822	1.372	No	No	GG
Qinghai	320	Female	40	Tibetan	5.617	4.014	No	No	GG
Qinghai	321	Female	41	Tibetan	3.570	91.312	No	No	GT
Qinghai	322	Female	41	Tibetan	1.593	6.395	No	No	GG
Qinghai	323	Female	41	Tibetan	2.755	12.001	Yes	Mild	GG
Qinghai	324	Female	41	Tibetan	0.730	5.305	No	No	GG
Qinghai	325	Female	42	Tibetan	3.506	24.764	Yes	Mild	GG
Qinghai	326	Male	42	Tibetan	1.275	7.034	Yes	Mild	GG
Qinghai	327	Male	42	Tibetan	1.816	5.639	No	No	GG
Qinghai	328	Male	42	Tibetan	2.607	0.907	No	No	GG
Qinghai	329	Female	42	Tibetan	5.713	9.206	No	No	GG
Qinghai	330	Female	42	Tibetan	1.948	10.058	Yes	Mild	GG
Qinghai	331	Male	42	Tibetan	7.034	23.721	No	No	GG
Qinghai	332	Male	42	Tibetan	0.428	5.641	Yes	Mild	GG
Qinghai	333	Male	42	Tibetan	0.474	3.380	Yes	Mild	GG
Qinghai	334	Female	43	Tibetan	4.316	8.593	No	No	GT
Qinghai	335	Male	43	Tibetan	1.817	5.202	No	No	GT
Qinghai	336	Female	43	Tibetan	2.375	5.959	No	No	GT

Qinghai	337	Female	43	Tibetan	0.914	5.301	Yes	Mild	GG
Qinghai	338	Female	43	Tibetan	2.352	10.808	No	No	GG
Qinghai	339	Male	43	Tibetan	5.535	10.246	Yes	Mild	GG
Qinghai	340	Male	43	Tibetan	5.986	10.859	No	No	GG
Qinghai	341	Male	43	Tibetan	6.230	11.190	Yes	Mild	GG
Qinghai	342	Female	43	Tibetan	2.587	9.863	No	No	GG
Qinghai	343	Male	43	Tibetan	2.661	6.347	No	No	GT
Qinghai	344	Female	44	Tibetan	1.209	4.376	No	No	GG
Qinghai	345	Female	44	Tibetan	0.746	3.749	No	No	GT
Qinghai	346	Male	44	Tibetan	3.512	7.502	No	No	GG
Qinghai	347	Female	44	Tibetan	1.336	4.549	No	No	GG
Qinghai	348	Male	44	Tibetan	2.192	22.656	No	No	GG
Qinghai	349	Female	44	Tibetan	2.802	10.178	No	No	GG
Qinghai	350	Female	44	Tibetan	2.399	8.335	No	No	GG
Qinghai	351	Male	44	Tibetan	3.561	2.507	No	No	GG
Qinghai	352	Male	44	Tibetan	6.835	12.011	Yes	Mild	GG
Qinghai	353	Female	45	Tibetan	1.747	0.605	Yes	Mild	GG
Qinghai	354	Male	45	Tibetan	3.822	10.821	No	No	GG
Qinghai	355	Female	45	Tibetan	1.406	0.620	No	No	GG
Qinghai	356	Male	45	Tibetan	4.151	8.272	Yes	Moderate	GG
Qinghai	357	Male	45	Tibetan	1.603	1.750	Yes	Moderate	GG
Qinghai	358	Female	45	Tibetan	1.602	7.291	No	No	GG
Qinghai	359	Male	45	Tibetan	4.683	9.090	Yes	Mild	GG
Qinghai	360	Male	46	Tibetan	3.518	7.510	Yes	Moderate	GG
Qinghai	361	Male	46	Tibetan	3.450	7.650	No	No	GG
Qinghai	362	Male	46	Tibetan	2.286	5.838	No	No	GT

Qinghai	363	Female	46	Tibetan	3.035	2.305	Yes	Mild	GG
Qinghai	364	Male	46	Tibetan	1.735	5.091	No	No	GG
Qinghai	365	Female	46	Tibetan	1.406	2.434	Yes	Mild	GT
Qinghai	366	Female	46	Tibetan	0.761	5.121	No	No	GG
Qinghai	367	Female	47	Tibetan	1.675	14.313	No	No	GG
Qinghai	368	Female	47	Tibetan	0.814	5.305	No	No	GG
Qinghai	369	Male	47	Tibetan	3.561	5.392	No	No	GG
Qinghai	370	Male	47	Tibetan	5.513	10.217	No	No	GG
Qinghai	371	Female	47	Tibetan	5.935	10.790	No	No	GG
Qinghai	372	Female	47	Tibetan	5.375	8.223	No	No	GG
Qinghai	373	Female	48	Tibetan	2.997	14.051	Yes	Moderate	GG
Qinghai	374	Male	48	Tibetan	0.524	1.410	Yes	Mild	GT
Qinghai	375	Female	48	Tibetan	2.735	7.051	Yes	Mild	GG
Qinghai	376	Female	48	Tibetan	4.710	5.378	No	No	GG
Qinghai	377	Female	48	Tibetan	0.730	3.727	No	No	GG
Qinghai	378	Male	48	Tibetan	2.506	8.223	No	No	GT
Qinghai	379	Female	48	Tibetan	5.521	9.581	No	No	GG
Qinghai	380	Female	49	Tibetan	2.305	5.864	Yes	Severe	GG
Qinghai	381	Male	49	Tibetan	2.351	10.250	Yes	Mild	GG
Qinghai	382	Male	49	Tibetan	4.014	7.389	No	No	GG
Qinghai	383	Female	49	Tibetan	1.559	2.405	No	No	GG
Qinghai	384	Male	49	Tibetan	1.881	5.288	No	No	GG
Qinghai	385	Female	49	Tibetan	4.138	11.217	Yes	Mild	GT
Qinghai	386	Male	49	Tibetan	8.029	37.381	Yes	Mild	GG
Qinghai	387	Male	49	Tibetan	1.753	10.969	No	No	GG
Qinghai	388	Male	50	Tibetan	1.687	5.026	No	No	GG

Qinghai	389	Male	50	Tibetan	1.209	4.376	No	No	GT
Qinghai	390	Male	50	Tibetan	4.568	8.935	No	No	GG
Qinghai	391	Male	50	Tibetan	4.080	8.273	No	No	GG
Qinghai	392	Male	50	Tibetan	1.152	4.300	No	No	GG
Qinghai	393	Male	50	Tibetan	4.612	8.994	Yes	Severe	GG
Qinghai	394	Female	50	Tibetan	3.476	5.455	No	No	GG
Qinghai	395	Female	50	Tibetan	3.242	7.135	No	No	GG
Qinghai	396	Male	50	Tibetan	1.788	10.742	No	No	GG
Qinghai	397	Female	50	Tibetan	1.193	4.356	No	No	GG
Qinghai	398	Male	50	Tibetan	0.958	15.286	No	No	GG
Qinghai	399	Female	50	Tibetan	1.666	2.140	No	No	GG
Qinghai	400	Male	50	Tibetan	1.540	1.751	No	No	GG
Qinghai	401	Male	50	Tibetan	3.816	4.510	No	No	GG
Qinghai	402	Female	50	Tibetan	2.802	6.539	Yes	Mild	GG
Qinghai	403	Male	50	Tibetan	4.015	8.184	Yes	Mild	GG
Qinghai	404	Female	51	Tibetan	1.626	9.841	No	No	GT
Qinghai	405	Female	51	Tibetan	1.889	9.349	Yes	Mild	GG
Qinghai	406	Male	51	Tibetan	3.502	1.485	Yes	Mild	GT
Qinghai	407	Female	51	Tibetan	2.172	21.132	No	No	GG
Qinghai	408	Female	51	Tibetan	1.881	5.288	No	No	GG
Qinghai	409	Female	51	Tibetan	1.667	4.998	Yes	Mild	GG
Qinghai	410	Male	51	Tibetan	0.997	7.352	Yes	Mild	GG
Qinghai	411	Female	51	Tibetan	2.587	6.247	No	No	GG
Qinghai	412	Female	51	Tibetan	0.639	3.604	No	No	GG
Qinghai	413	Female	52	Tibetan	1.957	5.391	No	No	GG
Qinghai	414	Female	52	Tibetan	1.842	5.235	No	No	GG

Qinghai	415	Female	52	Tibetan	1.851	12.301	Yes	Mild	GG
Qinghai	416	Female	52	Tibetan	0.986	1.672	No	No	GG
Qinghai	417	Female	52	Tibetan	1.139	2.613	No	No	GG
Qinghai	418	Male	52	Tibetan	1.540	4.826	No	No	GG
Qinghai	419	Male	52	Tibetan	4.178	7.310	No	No	GG
Qinghai	420	Male	52	Tibetan	4.057	8.242	No	No	GG
Qinghai	421	Female	53	Tibetan	1.509	4.785	No	No	GG
Qinghai	422	Female	53	Tibetan	1.583	0.088	Yes	Moderate	GG
Qinghai	423	Male	53	Tibetan	1.591	4.896	Yes	Moderate	GG
Qinghai	424	Female	53	Tibetan	4.526	8.877	Yes	Mild	GG
Qinghai	425	Male	54	Tibetan	1.665	4.996	Yes	Mild	GG
Qinghai	426	Male	54	Tibetan	2.107	5.595	Yes	Moderate	GG
Qinghai	427	Male	54	Tibetan	1.420	0.432	No	No	GG
Qinghai	428	Female	55	Tibetan	2.085	1.025	No	No	GG
Qinghai	429	Male	55	Tibetan	2.256	0.777	No	No	GG
Qinghai	430	Female	55	Tibetan	1.368	15.286	No	No	GG
Qinghai	431	Female	55	Tibetan	0.668	0.314	Yes	Mild	GT
Qinghai	432	Male	55	Tibetan	5.066	34.423	No	No	GG
Qinghai	433	Male	55	Tibetan	6.945	9.779	No	No	GG
Qinghai	434	Male	55	Tibetan	3.064	6.894	No	No	GG
Qinghai	435	Female	55	Tibetan	3.941	6.175	No	No	GG
Qinghai	436	Male	56	Tibetan	3.813	7.910	Yes	Mild	GG
Qinghai	437	Male	56	Tibetan	1.361	1.098	No	No	GG
Qinghai	438	Male	56	Tibetan	1.406	0.823	No	No	GG
Qinghai	439	Male	56	Tibetan	2.444	6.052	Yes	Mild	GG
Qinghai	440	Male	56	Tibetan	2.464	6.080	No	No	GG

Qinghai	441	Female	56	Tibetan	1.732	5.087	No	No	GG
Qinghai	442	Female	56	Tibetan	3.939	8.081	No	No	GG
Qinghai	443	Male	56	Tibetan	1.010	4.394	No	No	GG
Qinghai	444	Male	56	Tibetan	1.219	2.996	Yes	Mild	GG
Qinghai	445	Female	56	Tibetan	5.310	10.061	Yes	Mild	GG
Qinghai	446	Female	57	Tibetan	3.502	4.719	No	No	GG
Qinghai	447	Female	57	Tibetan	1.753	1.281	Yes	Mild	GG
Qinghai	448	Female	57	Tibetan	3.943	20.750	No	No	GG
Qinghai	449	Male	57	Tibetan	0.845	5.353	Yes	Mild	GG
Qinghai	450	Male	57	Tibetan	2.578	9.358	Yes	Moderate	GG
Qinghai	451	Male	58	Tibetan	2.067	5.541	No	No	GG
Qinghai	452	Male	58	Tibetan	1.406	2.254	No	No	GG
Qinghai	453	Female	58	Tibetan	4.857	9.327	No	No	GG
Qinghai	454	Female	58	Tibetan	7.491	4.536	No	No	GG
Qinghai	455	Female	58	Tibetan	2.267	14.504	No	No	GG
Qinghai	456	Male	58	Tibetan	2.267	2.656	No	No	GG
Qinghai	457	Female	58	Tibetan	5.642	15.082	No	No	GG
Qinghai	458	Female	58	Tibetan	2.130	11.717	Yes	Moderate	GG
Qinghai	459	Male	58	Tibetan	2.785	17.881	Yes	Severe	GG
Qinghai	460	Female	59	Tibetan	6.067	10.968	Yes	Mild	GG
Qinghai	461	Female	59	Tibetan	2.998	6.805	No	No	GT
Qinghai	462	Male	59	Tibetan	2.928	2.689	Yes	Mild	GG
Qinghai	463	Female	59	Tibetan	3.288	7.197	No	No	GG
Qinghai	464	Female	59	Tibetan	2.388	9.144	No	No	GG
Qinghai	465	Female	59	Tibetan	1.539	4.825	No	No	GG
Qinghai	466	Female	59	Tibetan	5.886	8.871	Yes	Severe	GG

Qinghai	467	Male	60	Tibetan	1.402	4.638	Yes	Mild	GT
Qinghai	468	Female	60	Tibetan	2.632	6.308	Yes	Mild	GG
Qinghai	469	Male	60	Tibetan	1.801	11.854	Yes	Moderate	GG
Qinghai	470	Male	60	Tibetan	4.226	8.470	Yes	Mild	GG
Qinghai	471	Male	60	Tibetan	2.478	6.099	Yes	Mild	GG
Qinghai	472	Male	60	Tibetan	1.127	2.668	Yes	Severe	GG
Qinghai	473	Female	60	Tibetan	2.165	4.487	No	No	GG
Qinghai	474	Male	60	Tibetan	7.641	3.802	Yes	Mild	GG
Qinghai	475	Female	60	Tibetan	2.671	1.690	Yes	Mild	GG
Qinghai	476	Female	61	Tibetan	0.535	11.910	No	No	GG
Qinghai	477	Male	61	Tibetan	2.351	5.926	Yes	Moderate	GG
Qinghai	478	Male	61	Tibetan	6.553	11.627	Yes	Moderate	GG
Qinghai	479	Female	61	Tibetan	1.126	0.274	No	No	GG
Qinghai	480	Female	61	Tibetan	4.865	9.933	No	No	GG
Qinghai	481	Male	61	Tibetan	1.331	11.995	Yes	Moderate	GG
Qinghai	482	Male	61	Tibetan	3.807	11.430	Yes	Moderate	GG
Qinghai	483	Female	61	Tibetan	2.224	2.578	Yes	Mild	GG
Qinghai	484	Male	61	Tibetan	4.320	8.598	Yes	Mild	GG
Qinghai	485	Male	62	Tibetan	3.398	7.347	No	No	GG
Qinghai	486	Male	62	Tibetan	2.493	1.387	No	No	GG
Qinghai	487	Male	62	Tibetan	2.118	5.528	No	No	GG
Qinghai	488	Male	62	Tibetan	4.605	11.717	Yes	Mild	GG
Qinghai	489	Female	62	Tibetan	4.470	8.802	Yes	Moderate	GG
Qinghai	490	Male	62	Tibetan	2.528	6.033	Yes	Mild	GG
Qinghai	491	Female	62	Tibetan	2.898	19.478	Yes	Moderate	GG
Qinghai	492	Male	63	Tibetan	1.082	4.205	No	No	GG

Qinghai	493	Female	63	Tibetan	3.909	10.102	Yes	Moderate	GG
Qinghai	494	Female	63	Tibetan	1.358	4.579	No	No	GG
Qinghai	495	Male	63	Tibetan	4.526	8.877	Yes	Mild	GG
Qinghai	496	Male	63	Tibetan	10.679	38.913	Yes	Moderate	GG
Qinghai	497	Female	63	Tibetan	6.080	4.877	No	No	GG
Qinghai	498	Female	64	Tibetan	2.311	0.371	Yes	Moderate	GG
Qinghai	499	Male	64	Tibetan	1.122	4.259	No	No	GG
Qinghai	500	Female	64	Tibetan	0.369	3.238	Yes	Mild	GG
Qinghai	501	Female	64	Tibetan	1.490	4.758	No	No	GG
Qinghai	502	Female	64	Tibetan	4.918	9.409	No	No	GG
Qinghai	503	Female	64	Tibetan	2.640	6.318	Yes	Severe	GG
Qinghai	504	Female	64	Tibetan	5.946	27.977	Yes	Mild	GG
Qinghai	505	Female	65	Tibetan	1.465	0.686	Yes	Mild	GG
Qinghai	506	Male	65	Tibetan	3.293	7.204	Yes	Moderate	GG
Qinghai	507	Male	65	Tibetan	4.154	2.346	No	No	GG
Qinghai	508	Male	65	Tibetan	3.706	0.549	No	No	GG
Qinghai	509	Female	65	Tibetan	4.870	9.344	Yes	Mild	GG
Qinghai	510	Male	65	Tibetan	1.841	1.444	Yes	Severe	GG
Qinghai	511	Female	65	Tibetan	3.157	15.052	No	No	GG
Qinghai	512	Male	66	Tibetan	1.644	0.274	No	No	GG
Qinghai	513	Male	66	Tibetan	1.955	5.389	Yes	Severe	GG
Qinghai	514	Female	66	Tibetan	1.924	0.137	Yes	Moderate	GG
Qinghai	515	Male	66	Tibetan	3.154	0.823	No	No	GG
Qinghai	516	Male	66	Tibetan	3.379	10.344	No	No	GG
Qinghai	517	Female	66	Tibetan	4.951	36.480	Yes	Moderate	GG
Qinghai	518	Male	67	Tibetan	2.278	5.828	Yes	Moderate	GG

Qinghai	519	Female	67	Tibetan	2.348	11.336	Yes	Mild	GG
Qinghai	520	Female	68	Tibetan	2.045	1.904	No	No	GG
Qinghai	521	Male	68	Tibetan	2.113	5.604	No	No	GG
Qinghai	522	Male	68	Tibetan	4.422	8.736	Yes	Mild	GG
Qinghai	523	Female	68	Tibetan	1.368	18.229	No	No	GG
Qinghai	524	Male	68	Tibetan	2.679	6.284	Yes	Severe	GG
Qinghai	525	Female	68	Tibetan	1.032	2.757	No	No	GG
Qinghai	526	Male	68	Tibetan	1.915	5.334	Yes	Severe	GG
Qinghai	527	Female	69	Tibetan	2.366	34.483	Yes	Severe	GG
Qinghai	528	Male	69	Tibetan	1.763	49.353	Yes	Mild	GT
Qinghai	529	Female	69	Tibetan	1.602	11.040	Yes	Mild	GG
Qinghai	530	Male	69	Tibetan	2.085	1.134	Yes	Severe	GG
Qinghai	531	Male	69	Tibetan	3.040	4.779	No	No	GG
Qinghai	532	Male	70	Tibetan	2.669	1.904	Yes	Mild	GT
Qinghai	533	Male	70	Tibetan	5.493	5.008	Yes	Severe	GG
Qinghai	534	Male	70	Tibetan	2.636	0.301	Yes	Moderate	GG
Qinghai	535	Female	70	Tibetan	9.780	12.983	No	No	GG
Qinghai	536	Female	70	Tibetan	3.807	2.425	No	No	GG
Qinghai	537	Male	70	Tibetan	6.516	13.651	Yes	Severe	GT
Qinghai	538	Male	70	Tibetan	2.785	11.336	No	No	GG
Qinghai	539	Female	70	Tibetan	4.436	18.988	Yes	Severe	GG
Qinghai	540	Male	70	Tibetan	3.267	17.262	Yes	Moderate	GG
Qinghai	541	Male	70	Tibetan	2.870	6.630	Yes	Severe	GG
Qinghai	542	Male	72	Tibetan	4.261	12.646	Yes	Mild	GG
Qinghai	543	Female	72	Tibetan	5.986	17.133	Yes	Moderate	GG
Qinghai	544	Female	72	Tibetan	3.747	7.820	Yes	Severe	GT

Qinghai	545	Female	72	Tibetan	2.567	6.220	No	No	GG
Qinghai	546	Female	73	Tibetan	2.798	2.954	No	No	GG
Qinghai	547	Male	73	Tibetan	2.191	4.133	Yes	Mild	GG
Qinghai	548	Female	73	Tibetan	3.117	6.966	Yes	Mild	GG
Qinghai	549	Female	73	Tibetan	3.695	7.750	Yes	Severe	GG
Qinghai	550	Male	73	Tibetan	1.674	5.008	No	No	GG
Qinghai	551	Female	73	Tibetan	1.698	3.007	Yes	Moderate	GG
Qinghai	552	Female	73	Tibetan	9.051	4.539	Yes	Moderate	GG
Qinghai	553	Female	73	Tibetan	2.374	22.480	Yes	Mild	GG
Qinghai	554	Male	73	Tibetan	2.089	4.615	Yes	Severe	GG
Qinghai	555	Female	74	Tibetan	2.619	4.591	No	No	GG
Qinghai	556	Female	74	Tibetan	3.611	17.556	No	No	GG
Qinghai	557	Male	76	Tibetan	3.422	10.386	Yes	Mild	GG
Qinghai	558	Female	77	Tibetan	3.493	5.797	No	No	GG
Qinghai	559	Female	78	Tibetan	5.187	5.673	No	No	GG
Qinghai	560	Male	78	Tibetan	1.479	4.743	Yes	Severe	GG
Qinghai	561	Female	79	Tibetan	0.777	3.792	Yes	Moderate	GT
Qinghai	562	Female	80	Tibetan	2.172	8.640	Yes	Severe	GG
Qinghai	563	Female	80	Tibetan	2.279	5.196	No	No	GG
Qinghai	564	Female	81	Tibetan	7.252	12.576	No	No	GG
Qinghai	565	Female	82	Tibetan	2.785	7.970	Yes	Mild	GG
Qinghai	566	Male	84	Tibetan	2.927	6.709	Yes	Severe	GG
Qinghai	567	Female	86	Tibetan	3.034	2.036	Yes	Moderate	GG
Qinghai	568	Female	86	Tibetan	1.204	4.371	Yes	Severe	GG
Qinghai	569	Female	###	Tibetan	2.033	5.495	Yes	Mild	GG
Sinkiang	570	Female	29	Kazakh	2.710	13.260	No	No	GG

Sinkiang	571	Male	29	Kazakh	3.843	7.952	No	No	GT
Sinkiang	572	Female	33	Kazakh	5.666	10.424	No	No	GG
Sinkiang	573	Male	36	Kazakh	7.066	12.323	No	No	GT
Sinkiang	574	Female	36	Kazakh	10.742	10.831	No	No	GG
Sinkiang	575	Male	36	Kazakh	9.756	16.103	No	No	GG
Sinkiang	576	Female	36	Kazakh	2.068	1.749	Yes	Mild	GG
Sinkiang	577	Female	36	Kazakh	5.070	5.821	No	No	GG
Sinkiang	578	Female	36	Kazakh	2.140	5.640	No	No	GG
Sinkiang	579	Female	36	Kazakh	1.689	4.726	No	No	GT
Sinkiang	580	Male	36	Kazakh	1.963	4.174	No	No	GG
Sinkiang	581	Female	36	Kazakh	2.478	14.627	No	No	GG
Sinkiang	582	Female	36	Kazakh	2.327	3.242	No	No	GT
Sinkiang	583	Male	36	Kazakh	3.651	8.452	No	No	GT
Sinkiang	584	Female	36	Kazakh	9.887	5.176	No	No	TT
Sinkiang	585	Female	36	Kazakh	4.797	1.294	Yes	Mild	GG
Sinkiang	586	Female	36	Kazakh	1.689	7.839	Yes	Mild	GG
Sinkiang	587	Female	36	Kazakh	3.416	2.777	No	No	GT
Sinkiang	588	Female	36	Kazakh	1.663	7.702	No	No	GG
Sinkiang	589	Female	37	Kazakh	5.642	12.491	No	No	GT
Sinkiang	590	Female	37	Kazakh	1.754	8.008	No	No	GG
Sinkiang	591	Female	37	Kazakh	3.597	5.473	No	No	GG
Sinkiang	592	Female	37	Kazakh	3.867	37.168	No	No	GG
Sinkiang	593	Female	37	Kazakh	2.971	13.065	No	No	GG
Sinkiang	594	Male	37	Kazakh	3.867	2.748	No	No	GT
Sinkiang	595	Female	38	Kazakh	3.166	11.485	No	No	GG
Sinkiang	596	Female	38	Kazakh	5.426	6.550	No	No	TT

Sinkiang	597	Male	39	Kazakh	7.334	3.244	No	No	GG
Sinkiang	598	Female	40	Kazakh	3.556	3.199	No	No	GT
Sinkiang	599	Female	40	Kazakh	4.851	4.459	No	No	GT
Sinkiang	600	Male	40	Kazakh	2.796	4.459	No	No	GG
Sinkiang	601	Female	40	Kazakh	6.880	5.047	No	No	GG
Sinkiang	602	Female	40	Kazakh	3.843	10.256	No	No	TT
Sinkiang	603	Female	40	Kazakh	6.463	32.140	No	No	GT
Sinkiang	604	Male	40	Kazakh	6.880	40.175	Yes	Mild	GG
Sinkiang	605	Female	40	Kazakh	7.435	7.068	No	No	GG
Sinkiang	606	Female	40	Kazakh	5.865	6.442	No	No	GG
Sinkiang	607	Female	40	Kazakh	2.412	5.581	No	No	TT
Sinkiang	608	Female	40	Kazakh	2.755	4.848	No	No	GG
Sinkiang	609	Female	40	Kazakh	2.687	5.132	Yes	Mild	GG
Sinkiang	610	Female	41	Kazakh	6.618	4.618	Yes	Moderate	GG
Sinkiang	611	Male	41	Kazakh	3.843	24.946	No	No	GG
Sinkiang	612	Female	41	Kazakh	4.015	9.694	No	No	GG
Sinkiang	613	Male	41	Kazakh	4.850	2.588	No	No	GT
Sinkiang	614	Female	42	Kazakh	2.764	6.487	No	No	TT
Sinkiang	615	Female	42	Kazakh	2.085	1.553	Yes	Mild	GG
Sinkiang	616	Female	42	Kazakh	1.266	3.642	Yes	Mild	GG
Sinkiang	617	Male	42	Kazakh	4.495	12.959	Yes	Mild	GG
Sinkiang	618	Female	42	Kazakh	8.594	23.947	No	No	GG
Sinkiang	619	Female	43	Kazakh	3.421	4.064	No	No	GG
Sinkiang	620	Female	43	Kazakh	3.996	0.822	No	No	GT
Sinkiang	621	Female	43	Kazakh	3.917	8.052	No	No	TT
Sinkiang	622	Female	43	Kazakh	5.450	8.636	Yes	Mild	GG

Sinkiang	623	Female	43	Kazakh	4.489	20.076	Yes	Mild	GT
Sinkiang	624	Female	43	Kazakh	0.689	4.494	No	No	GG
Sinkiang	625	Female	43	Kazakh	2.424	3.642	Yes	Mild	GG
Sinkiang	626	Male	43	Kazakh	5.643	37.168	No	No	GG
Sinkiang	627	Female	43	Kazakh	1.419	11.038	No	No	GG
Sinkiang	628	Female	44	Kazakh	2.654	3.192	No	No	GT
Sinkiang	629	Male	44	Kazakh	1.963	5.595	No	No	GT
Sinkiang	630	Female	44	Kazakh	1.821	3.264	No	No	GT
Sinkiang	631	Male	45	Kazakh	2.597	10.795	Yes	Mild	GG
Sinkiang	632	Male	45	Kazakh	2.929	3.076	No	No	GG
Sinkiang	633	Female	45	Kazakh	3.934	7.594	No	No	GG
Sinkiang	634	Female	45	Kazakh	4.667	3.372	Yes	Mild	GG
Sinkiang	635	Female	45	Kazakh	4.851	6.532	Yes	Mild	GG
Sinkiang	636	Female	45	Kazakh	3.556	11.295	No	No	GG
Sinkiang	637	Male	45	Kazakh	3.291	44.556	No	No	GG
Sinkiang	638	Female	45	Kazakh	1.469	4.729	No	No	TT
Sinkiang	639	Female	45	Kazakh	6.315	11.305	Yes	Mild	GT
Sinkiang	640	Female	45	Kazakh	1.957	11.998	No	No	GG
Sinkiang	641	Female	45	Kazakh	6.577	11.660	No	No	GG
Sinkiang	642	Female	45	Kazakh	0.704	3.692	Yes	Mild	GG
Sinkiang	643	Female	45	Kazakh	1.119	3.244	No	No	GG
Sinkiang	644	Female	45	Kazakh	7.325	12.675	No	No	GG
Sinkiang	645	Female	45	Kazakh	3.640	0.068	No	No	GG
Sinkiang	646	Female	45	Kazakh	1.406	2.247	Yes	Mild	GG
Sinkiang	647	Female	45	Kazakh	2.085	1.988	No	No	GT
Sinkiang	648	Female	45	Kazakh	0.413	3.297	No	No	GT

Sinkiang	649	Female	45	Kazakh	1.150	1.994	No	No	GT
Sinkiang	650	Female	45	Kazakh	3.724	7.734	No	No	GG
Sinkiang	651	Female	45	Kazakh	2.060	3.361	Yes	Mild	GG
Sinkiang	652	Female	45	Kazakh	3.867	8.384	No	No	GG
Sinkiang	653	Female	45	Kazakh	2.243	7.755	No	No	GG
Sinkiang	654	Female	45	Kazakh	2.117	30.017	Yes	Mild	GG
Sinkiang	655	Female	45	Kazakh	1.754	13.951	No	No	GG
Sinkiang	656	Male	46	Kazakh	2.998	4.985	No	No	GG
Sinkiang	657	Male	46	Kazakh	3.843	16.617	No	No	GG
Sinkiang	658	Male	46	Kazakh	8.645	2.356	No	No	GG
Sinkiang	659	Male	46	Kazakh	5.043	2.491	Yes	Mild	GG
Sinkiang	660	Female	46	Kazakh	18.747	28.173	No	No	GG
Sinkiang	661	Female	46	Kazakh	3.421	0.754	No	No	GT
Sinkiang	662	Female	46	Kazakh	0.167	2.963	Yes	Mild	GT
Sinkiang	663	Female	46	Kazakh	3.244	1.495	Yes	Mild	GT
Sinkiang	664	Female	46	Kazakh	3.610	8.634	Yes	Mild	GT
Sinkiang	665	Female	46	Kazakh	1.821	3.916	No	No	GG
Sinkiang	666	Female	47	Kazakh	6.363	7.770	No	No	GT
Sinkiang	667	Female	47	Kazakh	4.702	5.914	No	No	GG
Sinkiang	668	Female	47	Kazakh	4.154	22.193	Yes	Mild	GG
Sinkiang	669	Female	47	Kazakh	1.942	5.371	No	No	GT
Sinkiang	670	Female	47	Kazakh	2.324	5.890	No	No	GT
Sinkiang	671	Female	47	Kazakh	1.406	0.967	No	No	GG
Sinkiang	672	Female	47	Kazakh	2.198	8.415	Yes	Mild	GG
Sinkiang	673	Female	47	Kazakh	2.654	8.634	No	No	GT
Sinkiang	674	Female	47	Kazakh	3.341	8.075	Yes	Mild	GG

Sinkiang	675	Female	47	Kazakh	1.509	9.583	No	No	GT
Sinkiang	676	Female	48	Kazakh	5.890	14.424	No	No	GG
Sinkiang	677	Male	48	Kazakh	1.514	14.086	No	No	GT
Sinkiang	678	Female	48	Kazakh	6.123	14.086	No	No	TT
Sinkiang	679	Female	48	Kazakh	7.907	5.313	Yes	Mild	GG
Sinkiang	680	Female	48	Kazakh	3.265	10.194	No	No	GT
Sinkiang	681	Male	48	Kazakh	3.934	10.239	Yes	Mild	GG
Sinkiang	682	Female	48	Kazakh	5.043	9.579	Yes	Mild	GG
Sinkiang	683	Female	48	Kazakh	0.821	3.851	Yes	Mild	GT
Sinkiang	684	Female	48	Kazakh	2.085	1.304	No	No	GT
Sinkiang	685	Male	48	Kazakh	2.054	2.790	No	No	GG
Sinkiang	686	Female	48	Kazakh	1.406	2.318	Yes	Mild	GG
Sinkiang	687	Male	48	Kazakh	1.821	8.415	Yes	Mild	GT
Sinkiang	688	Female	48	Kazakh	1.713	1.784	Yes	Mild	GT
Sinkiang	689	Female	48	Kazakh	3.085	6.634	No	No	GT
Sinkiang	690	Male	48	Kazakh	7.608	8.075	No	No	GG
Sinkiang	691	Male	48	Kazakh	10.687	9.694	Yes	Mild	GT
Sinkiang	692	Female	48	Kazakh	4.390	9.412	No	No	GG
Sinkiang	693	Male	49	Kazakh	0.771	3.783	No	No	GG
Sinkiang	694	Female	49	Kazakh	3.474	8.008	No	No	GG
Sinkiang	695	Male	49	Kazakh	2.818	10.333	No	No	GG
Sinkiang	696	Male	49	Kazakh	3.166	6.532	No	No	GG
Sinkiang	697	Female	49	Kazakh	3.724	4.066	No	No	TT
Sinkiang	698	Female	49	Kazakh	2.755	5.809	No	No	GT
Sinkiang	699	Female	50	Kazakh	7.606	7.320	No	No	GG
Sinkiang	700	Female	50	Kazakh	3.697	5.023	Yes	Mild	GG

Sinkiang	701	Male	50	Kazakh	3.329	7.254	No	No	GG
Sinkiang	702	Male	50	Kazakh	4.851	7.212	No	No	GG
Sinkiang	703	Male	50	Kazakh	6.930	53.777	No	No	GT
Sinkiang	704	Female	50	Kazakh	4.576	12.876	No	No	GG
Sinkiang	705	Male	50	Kazakh	5.062	4.923	No	No	TT
Sinkiang	706	Male	50	Kazakh	3.291	11.295	Yes	Mild	GG
Sinkiang	707	Male	50	Kazakh	2.508	41.699	Yes	Moderate	GG
Sinkiang	708	Male	50	Kazakh	5.957	22.476	No	No	GG
Sinkiang	709	Female	50	Kazakh	1.488	1.477	Yes	Mild	GG
Sinkiang	710	Male	50	Kazakh	0.934	4.004	No	No	GG
Sinkiang	711	Male	50	Kazakh	2.710	1.656	Yes	Mild	GG
Sinkiang	712	Male	50	Kazakh	1.296	8.956	No	No	GG
Sinkiang	713	Female	50	Kazakh	2.501	6.130	No	No	GT
Sinkiang	714	Male	50	Kazakh	7.779	8.634	No	No	GG
Sinkiang	715	Female	50	Kazakh	2.971	1.553	Yes	Mild	GG
Sinkiang	716	Female	50	Kazakh	1.963	4.805	No	No	GG
Sinkiang	717	Male	50	Kazakh	2.317	2.845	No	No	GT
Sinkiang	718	Male	51	Kazakh	3.769	5.023	Yes	Severe	GT
Sinkiang	719	Female	51	Kazakh	1.380	4.610	Yes	Mild	GT
Sinkiang	720	Female	51	Kazakh	1.133	4.189	No	No	GT
Sinkiang	721	Female	51	Kazakh	2.696	10.203	Yes	Mild	GT
Sinkiang	722	Female	51	Kazakh	1.399	1.994	Yes	Mild	GG
Sinkiang	723	Male	51	Kazakh	3.732	9.073	Yes	Mild	GG
Sinkiang	724	Male	51	Kazakh	1.566	4.924	No	No	GT
Sinkiang	725	Female	52	Kazakh	6.618	11.716	Yes	Mild	GT
Sinkiang	726	Female	52	Kazakh	1.509	4.838	No	No	GT

Sinkiang	727	Male	53	Kazakh	4.234	5.684	Yes	Mild	GT
Sinkiang	728	Male	53	Kazakh	8.645	17.830	No	No	GG
Sinkiang	729	Male	53	Kazakh	3.697	20.076	No	No	GG
Sinkiang	730	Male	53	Kazakh	1.790	4.222	No	No	GG
Sinkiang	731	Male	53	Kazakh	1.406	0.207	No	No	GT
Sinkiang	732	Male	53	Kazakh	4.169	7.667	Yes	Mild	GG
Sinkiang	733	Female	53	Kazakh	2.060	8.680	No	No	GT
Sinkiang	734	Female	53	Kazakh	1.689	5.892	No	No	GG
Sinkiang	735	Male	53	Kazakh	1.506	6.079	Yes	Mild	GG
Sinkiang	736	Male	54	Kazakh	3.166	1.598	No	No	GG
Sinkiang	737	Female	54	Kazakh	10.543	23.166	No	No	GG
Sinkiang	738	Female	54	Kazakh	3.045	10.841	Yes	Mild	GG
Sinkiang	739	Male	54	Kazakh	3.502	7.488	No	No	GG
Sinkiang	740	Female	54	Kazakh	2.818	9.966	Yes	Mild	GG
Sinkiang	741	Female	54	Kazakh	2.243	1.812	No	No	GG
Sinkiang	742	Female	54	Kazakh	3.867	3.925	Yes	Moderate	GG
Sinkiang	743	Female	54	Kazakh	5.426	14.087	No	No	GG
Sinkiang	744	Male	55	Kazakh	5.101	15.614	No	No	GG
Sinkiang	745	Male	55	Kazakh	11.394	8.052	Yes	Mild	GT
Sinkiang	746	Male	55	Kazakh	5.666	15.614	No	No	GG
Sinkiang	747	Female	55	Kazakh	2.710	3.090	No	No	GG
Sinkiang	748	Male	55	Kazakh	1.839	7.712	Yes	Mild	GG
Sinkiang	749	Female	55	Kazakh	3.421	2.326	No	No	GT
Sinkiang	750	Female	55	Kazakh	3.085	5.905	No	No	GT
Sinkiang	751	Male	55	Kazakh	1.116	4.838	Yes	Mild	GG
Sinkiang	752	Female	56	Kazakh	2.593	2.718	Yes	Moderate	GG

Sinkiang	753	Female	56	Kazakh	3.640	6.634	No	No	GG
Sinkiang	754	Male	56	Kazakh	6.363	0.904	Yes	Mild	GG
Sinkiang	755	Male	56	Kazakh	4.505	5.905	No	No	GG
Sinkiang	756	Female	56	Kazakh	1.844	5.239	Yes	Mild	GG
Sinkiang	757	Female	57	Kazakh	3.045	6.868	No	No	GT
Sinkiang	758	Male	57	Kazakh	4.154	12.876	No	No	GG
Sinkiang	759	Female	57	Kazakh	1.279	4.472	No	No	GG
Sinkiang	760	Male	57	Kazakh	2.952	6.742	Yes	Mild	GG
Sinkiang	761	Female	57	Kazakh	4.495	4.135	Yes	Mild	GG
Sinkiang	762	Female	57	Kazakh	3.329	1.886	No	No	GT
Sinkiang	763	Female	58	Kazakh	4.757	2.263	Yes	Mild	GG
Sinkiang	764	Female	58	Kazakh	2.753	0.874	No	No	GG
Sinkiang	765	Female	58	Kazakh	2.929	5.672	No	No	GG
Sinkiang	766	Female	58	Kazakh	1.769	5.011	No	No	GG
Sinkiang	767	Male	58	Kazakh	3.626	16.889	Yes	Mild	GG
Sinkiang	768	Male	58	Kazakh	4.541	13.566	No	No	GG
Sinkiang	769	Female	58	Kazakh	4.851	9.319	No	No	GG
Sinkiang	770	Female	58	Kazakh	5.043	26.257	No	No	GG
Sinkiang	771	Female	58	Kazakh	1.116	2.188	No	No	TT
Sinkiang	772	Female	58	Kazakh	2.904	6.381	No	No	GG
Sinkiang	773	Male	58	Kazakh	4.015	1.627	No	No	GG
Sinkiang	774	Female	59	Kazakh	3.626	7.504	Yes	Mild	GG
Sinkiang	775	Female	59	Kazakh	1.518	4.797	Yes	Mild	GG
Sinkiang	776	Female	60	Kazakh	3.153	10.034	No	No	GT
Sinkiang	777	Male	60	Kazakh	2.956	6.739	Yes	Severe	GG
Sinkiang	778	Male	60	Kazakh	2.929	5.428	Yes	Moderate	GT

Sinkiang	779	Male	60	Kazakh	11.037	7.287	Yes	Mild	GT
Sinkiang	780	Female	60	Kazakh	4.594	3.786	No	No	GT
Sinkiang	781	Female	60	Kazakh	6.744	29.267	Yes	Mild	GT
Sinkiang	782	Female	60	Kazakh	3.843	7.952	No	No	GG
Sinkiang	783	Female	60	Kazakh	3.421	6.532	No	No	GG
Sinkiang	784	Male	60	Kazakh	5.620	5.033	Yes	Mild	GT
Sinkiang	785	Male	60	Kazakh	0.374	3.244	No	No	GG
Sinkiang	786	Female	60	Kazakh	2.083	8.483	No	No	GG
Sinkiang	787	Female	60	Kazakh	2.039	1.553	No	No	GG
Sinkiang	788	Male	60	Kazakh	1.551	3.882	Yes	Moderate	GG
Sinkiang	789	Female	61	Kazakh	4.048	1.204	No	No	GG
Sinkiang	790	Male	62	Kazakh	4.667	9.068	Yes	Mild	GG
Sinkiang	791	Male	62	Kazakh	3.141	6.525	Yes	Mild	GG
Sinkiang	792	Male	62	Kazakh	3.057	20.388	No	No	GT
Sinkiang	793	Female	62	Kazakh	8.035	13.639	No	No	GT
Sinkiang	794	Male	62	Kazakh	1.851	1.553	No	No	GT
Sinkiang	795	Male	62	Kazakh	1.116	3.576	No	No	GG
Sinkiang	796	Female	62	Kazakh	2.370	14.576	No	No	GG
Sinkiang	797	Female	62	Kazakh	11.379	18.176	Yes	Moderate	GT
Sinkiang	798	Male	63	Kazakh	7.729	13.224	Yes	Mild	GG
Sinkiang	799	Male	63	Kazakh	2.383	5.970	Yes	Mild	GG
Sinkiang	800	Male	63	Kazakh	4.668	1.920	Yes	Mild	GG
Sinkiang	801	Female	63	Kazakh	0.891	2.696	No	No	GG
Sinkiang	802	Male	64	Kazakh	4.318	2.430	Yes	Moderate	GG
Sinkiang	803	Female	64	Kazakh	2.764	1.131	No	No	GT
Sinkiang	804	Female	64	Kazakh	4.489	9.748	Yes	Mild	GT

Sinkiang	805	Female	64	Kazakh	6.802	1.553	No	No	GG
Sinkiang	806	Male	65	Kazakh	7.402	22.738	No	No	GG
Sinkiang	807	Female	65	Kazakh	2.732	12.988	Yes	Mild	GG
Sinkiang	808	Male	65	Kazakh	1.185	4.344	Yes	Mild	GT
Sinkiang	809	Male	65	Kazakh	2.638	17.997	Yes	Moderate	GG
Sinkiang	810	Female	65	Kazakh	2.321	17.409	No	No	GG
Sinkiang	811	Male	65	Kazakh	3.570	36.351	No	No	GG
Sinkiang	812	Female	65	Kazakh	1.453	5.754	No	No	GG
Sinkiang	813	Male	65	Kazakh	4.169	6.381	No	No	GG
Sinkiang	814	Female	65	Kazakh	1.754	4.358	Yes	Mild	GG
Sinkiang	815	Male	66	Kazakh	4.541	11.908	No	No	GG
Sinkiang	816	Male	66	Kazakh	6.075	3.365	Yes	Mild	GG
Sinkiang	817	Female	67	Kazakh	4.318	10.927	No	No	GT
Sinkiang	818	Male	67	Kazakh	2.764	3.912	No	No	GG
Sinkiang	819	Female	67	Kazakh	2.929	16.009	No	No	GT
Sinkiang	820	Male	67	Kazakh	1.406	0.120	Yes	Mild	GG
Sinkiang	821	Male	67	Kazakh	4.048	2.106	No	No	GG
Sinkiang	822	Male	67	Kazakh	1.713	7.191	Yes	Mild	GG
Sinkiang	823	Female	68	Kazakh	3.355	101.954	No	No	GT
Sinkiang	824	Female	68	Kazakh	8.035	4.177	No	No	TT
Sinkiang	825	Female	68	Kazakh	2.941	3.631	Yes	Mild	GG
Sinkiang	826	Female	68	Kazakh	9.027	7.002	No	No	GT
Sinkiang	827	Female	68	Kazakh	2.818	3.224	No	No	GT
Sinkiang	828	Female	68	Kazakh	3.724	4.400	Yes	Mild	GG
Sinkiang	829	Female	68	Kazakh	2.654	17.415	No	No	GG
Sinkiang	830	Female	69	Kazakh	2.066	6.357	No	No	GT

Sinkiang	831	Male	69	Kazakh	3.598	8.797	No	No	GT
Sinkiang	832	Female	70	Kazakh	4.104	3.341	Yes	Moderate	GG
Sinkiang	833	Female	70	Kazakh	6.744	12.367	No	No	GT
Sinkiang	834	Female	70	Kazakh	1.407	3.501	Yes	Mild	GT
Sinkiang	835	Male	70	Kazakh	0.506	1.739	No	No	GT
Sinkiang	836	Female	70	Kazakh	2.117	1.739	No	No	GT
Sinkiang	837	Male	70	Kazakh	2.861	14.822	No	No	GG
Sinkiang	838	Male	70	Kazakh	1.591	4.896	Yes	Mild	GG
Sinkiang	839	Female	70	Kazakh	2.755	4.537	No	No	GG
Sinkiang	840	Male	71	Kazakh	2.487	20.468	No	No	GG
Sinkiang	841	Female	71	Kazakh	1.615	10.759	No	No	GT
Sinkiang	842	Male	72	Kazakh	3.045	1.405	No	No	GT
Sinkiang	843	Male	72	Kazakh	1.861	6.634	Yes	Mild	GT
Sinkiang	844	Male	72	Kazakh	9.193	12.012	No	No	GG
Sinkiang	845	Female	72	Kazakh	4.495	20.349	No	No	GG
Sinkiang	846	Female	73	Kazakh	2.884	24.225	No	No	GT
Sinkiang	847	Male	73	Kazakh	1.879	5.637	No	No	GT
Sinkiang	848	Male	74	Kazakh	2.085	1.617	No	No	GG
Sinkiang	849	Female	74	Kazakh	4.154	5.164	Yes	Moderate	GT
Sinkiang	850	Male	74	Kazakh	3.586	3.882	Yes	Mild	GG
Sinkiang	851	Male	75	Kazakh	2.147	3.501	Yes	Severe	GT
Sinkiang	852	Male	77	Kazakh	4.385	6.154	No	No	GT
Sinkiang	853	Male	77	Kazakh	4.667	10.166	Yes	Mild	GT
Sinkiang	854	Male	77	Kazakh	3.203	4.922	Yes	Severe	GT
Sinkiang	855	Male	78	Kazakh	10.543	2.785	No	No	GG
Sinkiang	856	Female	78	Kazakh	1.467	4.728	No	No	GT

Sinkiang	857	Female	80	Kazakh	1.862	2.814	No	No	GG
Sinkiang	858	Male	80	Kazakh	1.159	4.309	Yes	Severe	GG
Sinkiang	859	Female	85	Kazakh	1.844	11.618	No	No	GT
